# Accurate Confidence and Bayesian Interval Estimation for Non-centrality Parameters and Effect Size Indices

**DOI:** 10.1007/s11336-022-09899-x

**Published:** 2023-02-01

**Authors:** Kaidi Kang, Megan T. Jones, Kristan Armstrong, Suzanne Avery, Maureen McHugo, Stephan Heckers, Simon Vandekar

**Affiliations:** 1 Department of Biostatistics, Vanderbilt University, 2525 West End Ave.,#1136, Nashville, TN 37203 USA; 2 Department of Psychiatry and Behavioral Sciences, Vanderbilt University Medical Center, Nashville, USA

**Keywords:** effect size, robust effect size index, confidence interval, credible interval, analysis of effect size, non-centrality parameter, bootstrap, bayesian bootstrap, non-central Chi-squared distribution, non-central F distribution

## Abstract

Reporting effect size index estimates with their confidence intervals (CIs) can be an excellent way to simultaneously communicate the strength and precision of the observed evidence. We recently proposed a robust effect size index (RESI) that is advantageous over common indices because it’s widely applicable to different types of data. Here, we use statistical theory and simulations to develop and evaluate RESI estimators and confidence/credible intervals that rely on different covariance estimators. Our results show (1) counter to intuition, the randomness of covariates reduces coverage for Chi-squared and F CIs; (2) when the variance of the estimators is estimated, the non-central Chi-squared and F CIs using the parametric and robust RESI estimators fail to cover the true effect size at the nominal level. Using the robust estimator along with the proposed nonparametric bootstrap or Bayesian (credible) intervals provides valid inference for the RESI, even when model assumptions may be violated. This work forms a unified effect size reporting procedure, such that effect sizes with confidence/credible intervals can be easily reported in an analysis of variance (ANOVA) table format.

**Supplementary Information:**

The online version contains supplementary material available at 10.1007/s11336-022-09899-x.

Effect size indices, as generally defined by Kelley & Preacher (2012), are *a quantitative reflection of the magnitude of some phenomenon that is used for the purpose of addressing a question of interest*. They are unaffected by the sample size and play an important role in power analyses, sample size planning and meta-analyses (Cohen, 1988; Chinn, 2000; Morris & DeShon, 2002). Given the recent criticism of the misuse and misinterpretation of null hypothesis significance testing by the American Statistical Association (ASA) (Wasserstein & Lazar, 2016; Wasserstein et al., 2019; Kafadar, 2021), there is an increasing interest in seeking alternatives for communicating study findings. Reporting effect size estimates with confidence intervals (CIs) can be an excellent way to simultaneously communicate the strength of evidence (sample size independent) as well as the confidence one should have in the evidence (sample size dependent). The American Psychological Association (APA) has also been calling for reporting of effect sizes and their CIs for almost three decades (American Psychological Association, 1994; Wilkinson and the Task Force on Statistical Inference, 1999, American Psychological Association, 2001, 2010), but they are still not routinely reported by psychological studies. Fritz et al. (2012) reviewed articles published in 2009 and 2010 in the *Journal of Experimental Psychology: General*, and noted that less than half of the articles they reviewed reported effect sizes and no article reported a confidence interval for an effect size. More recently, Amaral & Line (2021) found low reporting rate of effect sizes in 119,558 clinical or biomedical studies published between 2019 and 2020 and advocated greater emphasis on reporting them. The barriers stopping researchers from easily reporting effect sizes along with their CIs not only lie in their unfamiliarity with different effect size indices but also in the lack of guidance of how to correctly estimate the CI for a specific effect size index.

We recently proposed a robust effect size index (RESI) (Vandekar et al., 2020), which is a standardized parameter describing the deviation of the true parameter value from a reference value. It has several advantages over previously proposed indices (Hedges & Olkin, 1985; Cohen, 1988; Rosenthal, 1994; Zhang & Schoeps, 1997; Long & Freese, 2006) because (1) it is widely applicable to many types of data since it is constructed from M-estimators, which are generally defined; (2) it is robust to possible model misspecification as it can use a heteroskedasticity consistent covariance estimator; (3) it can accommodate the existence of nuisance covariates/parameters. We also proposed a simple consistent estimator for the RESI that is a function of the Chi-squared test statistic (Vandekar et al., 2020). The RESI is defined on the basis of the Wald test statistic—it is related to the non-centrality parameter (NCP) of the test statistic under the alternative hypothesis, therefore, it has the generality that it can be estimated in different types of data. Researchers can use the RESI to report their observed effect sizes regardless of the model. Furthermore, studies on the same scientific topic, but using different types of data can easily communicate their observed effect sizes without translating between different effect size indices. While the RESI can currently be used to report the strength of a finding through the RESI estimate, we did not establish a CI estimation procedure for the RESI, which quantifies the amount of certainty of the estimate.

The goal of this paper is to establish an accurate interval estimation procedure for the RESI and establish a framework for the analysis of effect sizes based on the RESI. Because the RESI is related to the NCP of the Chi-squared statistic, an intuitive approach is to use existing methods based on non-central distributions to construct intervals for the NCP of a noncentral Chi-squared or F distribution (Kent & Hainsworth, 1995; Steiger & Fouladi, 1997). Here, we use statistical theory and simulations to show that the Chi-squared CI provides low coverage for the NCP when the variance must be estimated. In fact, the coverage gets lower with increasing sample and effect size. Similarly, the F CI has decreasing coverage with increasing sample size when the robust covariance estimate is used. We use theory to show that this occurs because the variance of the estimators is not asymptotically equivalent to each other. As a solution, we propose bootstrap interval construction procedures and evaluate their coverage performance through simulations in various scenarios. We show the nonparametric bootstrap CI with the robust estimator of the RESI is generally accurate and applicable, even when model assumptions are violated. In addition, we propose a credible interval derived by using Bayesian bootstraps for the robust estimator as an alternative interval that has the Bayesian instead of Frequentist interpretation (Rubin, 1981). The Bayesian bootstrap simulates the posterior distribution of the true effect size using a non-informative Dirichlet prior distribution on the sampling weight of each observation and a non-informative flat improper prior on the RESI. The credible interval performs comparably to the nonparametric CI, except in very small sample sizes. By using RESI, the effect sizes and confidence/credible intervals can be easily reported in a coefficient table or analysis of variance (ANOVA) table format. We use this framework to study the effect of early psychosis and schizophrenia on relational memory. Our RESI R package is available on CRAN (https://cran.r-project.org/web/packages/RESI/index.html).

## Statistical Theory

### Estimators for the Robust Effect Size Index (RESI)

In this section, we define the RESI and describe three estimators for the parameter. Let $W = (W_1, \ldots , W_n)$ denote the full dataset, where $W_i$ is independent of $W_j$ for all $i\ne j$. Assume $\theta = (\alpha , \beta ) \in \Theta \subset \mathbb {R}^m$ are model parameters, where $\alpha \in \mathbb {R}^{m_0}$ is a vector of nuisance parameters, $\beta \in \mathbb {R}^{m_1}$ is a vector of target parameters, and $m = m_0 + m_1$. We assume $\Psi (\theta ; W) = n^{-1}\sum _{i=1}^n \psi (\theta ; W) \in \mathbb {R}$ is an estimating equation, where $\psi$ is a known function and $\Psi$ can be maximized to obtain the M-estimator ${\hat{\theta }}$

$$\begin{aligned} {\hat{\theta }} = \mathop {\mathrm{arg\,max}}\limits _{\theta ^* \in \Theta } \Psi (\theta ^*; W). \end{aligned}$$

If $\Psi$ is a log-likelihood function, then ${{\hat{\theta }}}$ corresponds to the maximum likelihood estimator. Denote the target value of the parameter $\theta = \arg \max _{\theta ^*}\mathbb {E}[\Psi (\theta ^*; W)]$.

We define the components of the asymptotic covariance of $\sqrt{n}({\hat{\theta }}-\theta )$:

$$\begin{aligned} {\textbf {J}}_{jk}(\theta )=&{} - \lim _{n\rightarrow \infty }\mathbb {E} \left[ \frac{\partial ^2 \Psi (\theta ^*, W)}{\partial \theta ^*_j \partial \theta ^*_k} \Big |_\theta \right] \\ {\textbf {K}}_{jk}(\theta )=&{} \lim _{n\rightarrow \infty } n \mathbb {E}\left[ \frac{\partial \Psi (\theta ^*, W)}{\partial \theta ^*_j} \frac{\partial \Psi (\theta ^*, W)}{\partial \theta ^*_k} \Big |_\theta \right] \end{aligned}$$

Under mild conditions (Van der Vaart, 2000; Vandekar et al., 2020), the variance of $\sqrt{n}({\hat{\theta }}-\theta )$ is

1 $$\begin{aligned} \Sigma _{\theta } = \textbf{J}(\theta )^{-1} \textbf{K}(\theta ) \textbf{J}(\theta )^{-1} \end{aligned}$$

If $\Psi$ is a correctly specified likelihood function, then $\textbf{J}(\theta ) = \textbf{K}(\theta )$, and the asymptotic covariance matrix of $\sqrt{n}({\hat{\theta }}-\theta )$ is $\textbf{J}(\theta )^{-1}$.

We defined the RESI from the test statistic for $H_0: \beta = \beta _0$, where $\beta _0$ is a vector-valued reference point. The RESI depends upon the reference value, $\beta _0$, so may seem not to conform to the general definition of effect size given by Kelley & Preacher (2012) at first, as it explicitly depends on the reference value. However, this is implicitly true of existing effect size indices as well. For example, Cohen’s *d* is defined as the standardized difference in the sample means between the two comparison groups, the reference point is implicitly taken to be zero. In the vast majority of cases for the RESI, the reference value, $\beta _0$, will be zero.

We previously suggested that the typical Wald-style statistic for the test of the null hypothesis follows a Chi-squared distribution with $m_1$ degrees of freedom and non-centrality parameter $n(\beta - \beta _0 )^T \Sigma _{\beta }^{-1}(\theta ) (\beta - \beta _0)$,

2 $$\begin{aligned} T^2 = n({\hat{\beta }} - \beta _0 )^T \Sigma _{\beta }^{-1}(\theta ) ({\hat{\beta }} - \beta _0) \sim \chi ^2_{m_1}\{ n(\beta - \beta _0 )^T \Sigma _{\beta }^{-1}(\theta ) (\beta - \beta _0) \}, \end{aligned}$$

where the ${\hat{\beta }}$ is the estimated value of $\beta$ and $\Sigma _{\beta }(\theta )$ is the asymptotic covariance matrix of $\sqrt{n}({\hat{\beta }}-\beta )$.

The RESI was defined as the square root of the component of the NCP that is due to the deviation of $\beta$ from the reference value,

$$\begin{aligned} S_{\beta } = \sqrt{(\beta - \beta _0 )^T \Sigma _{\beta }^{-1}(\theta ) (\beta - \beta _0)}. \end{aligned}$$

The estimator for the RESI is defined as (Vandekar et al., 2020)

3 $$\begin{aligned} {\hat{S}}_{\beta } = \left\{ \max \left[ 0, \frac{T^2 - m_1}{n} \right] \right\} ^{\frac{1}{2}} \end{aligned}$$

where $T^2$ is as defined in (2). This estimator was derived by setting the observed statistic $T^2$ equal to the expected value of the non-central Chi-squared distribution and solving for $S_\beta$

$$\begin{aligned} {\hat{S}}_{\beta }^2&= \frac{T^2 - m_1}{n}. \end{aligned}$$

Because $S_{\beta }$ must be nonnegative, the estimator (3) has lower mean square error (Neff & Strawderman, 1976; Kubokawa et al., 1993; Vandekar et al., 2020).

There are several ways of constructing the statistic $T^2$ and ${{\hat{S}}}_\beta$ through the estimation of $\Sigma _{\beta }({{\hat{\theta }}})$ (White, 1980; MacKinnon & White, 1985; Long & Ervin, 2000). First, the matrices $\textbf{J}$ and $\textbf{K}$ are estimated by

4 $$\begin{aligned} \hat{\textbf{J}}_{jk}({{\hat{\theta }}})&= - n^{-1}\sum _{i=1}^n \frac{\partial ^2 \psi (\theta ^*, W)}{\partial \theta ^*_j \partial \theta ^*_k} \Big | _{{{\hat{\theta }}}} \end{aligned}$$

5 $$\begin{aligned} \hat{\textbf{K}}({{\hat{\theta }}})&= n^{-1}\sum _{i=1}^n \frac{\partial \psi (\theta ^*, W)}{\partial \theta ^*_j} \frac{\partial \psi (\theta ^*, W)}{\partial \theta ^*_k} \Big |_{{\hat{\theta }}} \nonumber \\ {\hat{\Sigma }}({{\hat{\theta }}})&= \hat{\textbf{J}}({{\hat{\theta }}})^{-1} \hat{\textbf{K}}({{\hat{\theta }}}) \hat{\textbf{J}}({{\hat{\theta }}})^{-1} . \end{aligned}$$

Then we can estimate the RESI using three versions of test statistics:

1. **Oracle Test Statistics**: when the true covariance of $\sqrt{n}({\hat{\beta }}-\beta )$ is known,
  $$\begin{aligned} T_{(o)}^2 = n({\hat{\beta }} - \beta _0 )^T \Sigma _{\beta }^{-1}(\theta ) ({\hat{\beta }} - \beta _0), \end{aligned}$$
  where $\Sigma _{\beta }(\theta )$ is obtained from the block diagonal of (1) corresponding to $\beta$. This is called an “oracle” statistic because it depends on the true covariance matrix, which is not known in practice.
2. **Parametric Test Statistics**: when we believe the working model is correctly specified,
  $$\begin{aligned} T_{(p)}^2 = n({\hat{\beta }} - \beta _0 )^T \hat{\textbf{J}}_{\beta }({{\hat{\theta }}}) ({\hat{\beta }} - \beta _0), \end{aligned}$$
  where $\hat{\textbf{J}}_{\beta }({{\hat{\theta }}})$ is obtained from the block diagonal component of (4) corresponding to $\beta$.
3. **Robust Test Statistics:** when the working model is not assumed to be correct,
  $$\begin{aligned} T_{(r)}^2 = n({\hat{\beta }} - \beta _0)^T {\hat{\Sigma }}_{\beta }^{-1}({{\hat{\theta }}}) ({\hat{\beta }} - \beta _0), \end{aligned}$$
  where ${\hat{\Sigma }}_{\beta }({{\hat{\theta }}})$ is obtained from the block diagonal component of (5) corresponding to $\beta$.

With the oracle, parametric, or robust test statistics, we can derive the corresponding oracle, parametric or robust estimator for the RESI using (3). These different versions of the estimator for $S_\beta$ have different sampling distributions depending on which test statistic is used.

### CIs Based on Non-central Distributions Underestimate the Variance

To compare the sampling distributions of the oracle, parametric, and robust test statistics under the alternative, in this section, we compare the variance of the test statistics under linear regression analytically. When the covariance of $\sqrt{n}({\hat{\beta }}-\beta )$ is known, the test statistic follows the non-central Chi-squared distribution (2), where its variance depends on the true effect size. However, in practice, the covariance needs to be estimated. In this section, we will show that the large sample distribution of the test statistic deviates from the theoretical non-central Chi-squared distribution when an estimator for the covariance is used. As a result, the CIs constructed using Chi-squared distribution (Kent & Hainsworth, 1995) will fail to provide nominal coverage. This is in contrast to the null case, where the asymptotic distribution of the Chi-square statistic is valid whether or not $\Sigma _{\beta }(\theta )$ is known or estimated. To illustrate this problem here, we compare the asymptotic equivalence of the variance of the oracle, parametric, and robust estimators in a linear model. Two functions *f*(*n*), *g*(*n*) are said to be asymptotically equivalent if $\lim _{n \rightarrow \infty } \frac{f(n)}{g(n)} = 1$. We need asymptotic equivalence here, because a standard asymptotic approach cannot be used; under the alternative, the mean and variance of the test statistics depend on the sample size.

Throughout this section, we assume $Y = X\beta + \epsilon$, where *X* is full rank and $\epsilon _i$ are independent with mean 0 and variance $\sigma ^2_i$. Note that $\sigma ^2_i$ may not be equal. The ordinary least squares (OLS) estimator of $\beta$ is ${\hat{\beta }}_{OLS} = (X^T X)^{-1} X^T Y$.

*Oracle Estimator: * Assuming known covariance, the test statistic (2) is approximately central Chi-squared by the central limit theorem under the null. Under the alternative, a similar argument suggests its approximation to a non-central Chi-squared distribution.

$$\begin{aligned} T_{(o)}^2 \sim \chi ^2_{m_1}( nS^2_{\beta } ). \end{aligned}$$

The expectation and variance of the oracle test statistics are:

6 $$\begin{aligned} \mathbb {E}T_{(o)}^2&= m_1 + nS^2_{\beta } \nonumber \\ \textrm{Var}(T_{(o)}^2)&= 2(m_1 + 2nS^2_{\beta }). \end{aligned}$$

Thus, the estimator (3) is consistent and the variance of $T_{(o)}$ is linear in the sample size.

*Parametric Estimator: * Assuming homoskedasticity (i.e., $\sigma ^2_i = \sigma ^2$, $\forall i$) and normality of *Y* makes finding the distribution of the test statistic tractable. The covariance matrix of ${\hat{\beta }}_{OLS}$ can be estimated as $(X^T X)^{-1} {\hat{\sigma }}^2$, where ${\hat{\sigma }}^2 = (n-m)^{-1} Y^T (I - H) Y$ and *H* is the hat matrix $H = X (X^TX)^{-1} X^T$. In this situation, the parametric version of test statistic for $H_0:\beta = \beta _0$ is

7 $$\begin{aligned} T_{(p)}^2&= n ({\hat{\beta }}_{OLS} - \beta _0)^T {\hat{\Sigma }_{\beta }^{-1}} ({\hat{\beta }}_{OLS} - \beta _0) \nonumber \\&= {\hat{\sigma }}^{-2} ({\hat{\beta }}_{OLS} - \beta _0)^T X^T X ({\hat{\beta }}_{OLS} - \beta _0) \end{aligned}$$

Additional variability is introduced into the test statistic when plugging in the estimated covariance of ${\hat{\beta }}_{OLS}$ and, consequently, the parametric test statistic does not follow the non-central Chi-squared distribution (2). For linear regression models, it can be shown that the test statistic divided by its own degrees of freedom $m_1$ follows the non-central F distribution (see Appendix)

8 $$\begin{aligned} T_{(p)}^2 / m_1 \sim F(m_1, n - m; nS^2_{\beta }). \end{aligned}$$

By plugging the parametric test statistic (7) into (3), we can derive a parametric version of the estimator for the RESI. The expectation and variance of the parametric test statistic are derived from the moments of the F distribution,

$$\begin{aligned} \mathbb {E}T_{(p)}^2&= \frac{(n-m)(m_1 + nS^2_{\beta })}{n-m - 2}\\ \textrm{Var}\left( T_{(p)}^2 \right)&= 2 \frac{(m_1 + nS^2_{\beta })^2 + (m_1 + 2nS^2_{\beta })(n-m-2)}{(n-m-2)^2 (n-m-4)}\left( n-m \right) ^2 \end{aligned}$$

$\mathbb {E}T_{(p)}^2$ and $\textrm{Var} \left( T_{(p)}^2 \right)$ are asymptotically equivalent to

9 $$\begin{aligned} \mathbb {E}T_{(p)}^2&\simeq m_1 + nS^2_{\beta }\nonumber \\ \textrm{Var} \left( T_{(p)}^2 \right)&\simeq 2(m_1 + 2nS^2_{\beta }) + 2\left( 2 m_1 S^2_{\beta } + nS^4_{\beta } \right) \nonumber \\&= \textrm{Var} \left( T_{(o)}^2 \right) + 2\left( 2 m_1 S^2_{\beta } + nS^4_{\beta } \right) . \end{aligned}$$

This parametric test statistic, which is the simplest unknown variance case, has a variance that is not asymptotically equal to the variance of the oracle test statistic, which follows the non-central Chi-squared distribution. The consequence of this fact is that a CI using the Chi-squared distribution will have lower than nominal coverage for the NCP and effect sizes that are a function of the NCP. In fact, the CI will become increasingly inaccurate as the effect size or sample size get larger. We demonstrate this with simulations in Sect. 4.

*Robust Estimator: * When there is a suspected unknown heteroskedasticity, a robust version of covariance estimator of ${\hat{\beta }}_{OLS}$ can be applied instead,

$$\begin{aligned} \hat{\Sigma }_{\beta _{\textrm{OLS} } } = (X^T X)^{-1}X^T {\hat{G}} X (X^T X)^{-1} \end{aligned}$$

where ${\hat{G}}_{ij} = \frac{e_i^2}{(1 - h_i)^2}$ if $i = j$ and ${\hat{G}}_{ij} = 0$ if $i \ne j$, $e_i = Y_i - X_i{\hat{\beta }}$ is the *i*th residual, and $h_i$ is the *i*-th element on the diagonal of the hat matrix *H*, i.e., $h_i = x_i (X^TX)^{-1} x_i^T$ (Long & Ervin, 2000). Many versions of this robust “sandwich" covariance estimator have been proposed, this version is discussed by Long & Ervin (2000) as a jackknife approximation.

The robust version of the test statistic in this case is

10 $$\begin{aligned} T_{(r)}^2&= n ({\hat{\beta }}_\textrm{OLS} - \beta _0)^T {\hat{\Sigma }}_{\beta }^{-1}({\hat{\beta }}_\textrm{OLS} - \beta _0) \nonumber \\&= ({\hat{\beta }}_\textrm{OLS} - \beta _0)^T (X^T X) (X^T {\hat{G}} X)^{-1} (X^T X) ({\hat{\beta }}_\textrm{OLS} - \beta _0) . \end{aligned}$$

Then the robust version of the estimator for RESI can be constructed by plugging (10) into (3).

Similar to the situation of parametric test statistic, the covariance of parameter estimator ${\hat{\beta }}_\textrm{OLS}$ is estimated. The estimator is still consistent

$$\begin{aligned} \mathbb {E}T_{(r)}^2&= \textrm{tr} \left( \left( n^{-1} \hat{\Sigma }_{\beta } \right) ^{-1} n^{-1}\Sigma _{\beta } \right) + n(\mathbb {E}{\hat{\beta }}_\textrm{OLS} - \beta _0)^T {\hat{\Sigma }}_{\beta }^{-1} (\mathbb {E}{\hat{\beta }}_\textrm{OLS} - \beta _0) \\&\simeq \textrm{tr} \left( \Sigma _{\beta }^{-1} \Sigma _{\beta } \right) + n(\beta - \beta _0)^T \Sigma _{\beta }^{-1} (\beta - \beta _0) \\&= \textrm{tr} \left( \textbf{I}_{m_1} \right) + nS^2 \\&= m_1 + nS^2 = \mathbb {E}T^2_{(o)}, \end{aligned}$$

but using the robust covariance estimator increases variance of the robust test statistic above the non-central Chi-squared and the F distributions. We found an analytical form for the variance of $T_{(r)}^2$ to be intractable, so estimated it using simulations.

**Fig. 1:**
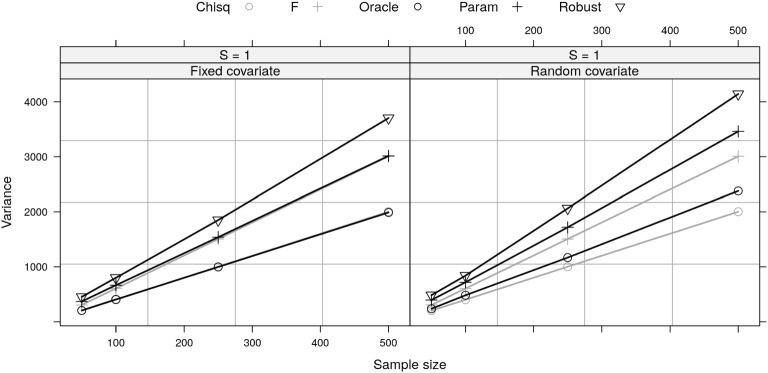
A comparison of simulated variance of the three test statistics from a simple linear regression model with a binary covariate and assumptions of homoskedasticity and symmetric errors. The grey lines are the variance of theoretical non-central F or Chi-squared distribution using formulas (6) and (9). The black lines are the simulated variance of test statistics with random or fixed covariate. With fixed covariate, the variance of oracle and parametric statistics equals theoretical variance of the non-central Chi-squared or F distribution, respectively. With random covariates, the true variance of the test statistic deviates from the theoretical value. *S* denotes the RESI; Chisq and F denote the non-central Chi-squared and F distributions, respectively; Oracle, Param and Robust denote the oracle, parametric and robust test statistics, respectively.

Figure 1 shows the simulated variance of each test statistic as a linear function of *n*. When the effect size is large (e.g., $S = 1$) and with a fixed covariate, the variance of oracle and parametric statistics equals the variance of non-central Chi-squared and F distributions, respectively. This is expected since these are the correct distributions of these two statistics, respectively (Sect. 1.2). Because the test statistic using the robust covariance has larger variance than the oracle and parametric statistics, the robust test statistic is not approximately non-central Chi-squared or non-central F distributed. This implies that the CIs constructed using either of these two distributions will not have accurate coverage for the robust RESI estimator and that the coverage will get worse with increasing effect size or sample size. When the covariate is random (Fig. 1), the variance of all three statistics further grows, and both of the oracle and parametric statistics deviate from the non-central Chi-squared and F distributions. This implies that when the design matrix is random (such as in observational studies), neither of these two distributions will produce CIs with nominal coverage for any of these estimators. While we only studied linear models in this section, we expect that the robust test statistic will not follow the Chi-squared or F distribution under the alternative in general.

## Methods

### Intervals for RESI

Here, we discuss several potential procedures to construct intervals using non-central Chi-squared and F distributions and bootstrapping.

#### Theoretical Methods: Using Non-central Distributions

As discussed in the previous section, the test statistic may follow a non-central Chi-squared or F distribution with a non-centrality parameter (NCP) not equal to 0. Since the squared RESI was defined as the NCP divided by sample size *n*, there is a one-to-one relationship between the NCP and RESI. If a CI for the NCP can be constructed, the CI for RESI can be derived from the CI for NCP. The confidence interval construction for NCP has been discussed for non-central Chi-squared and F distributions (Kent & Hainsworth, 1995; Steiger & Fouladi, 1997).

Suppose the test statistics from the sample $T_\textrm{obs}^2$ is observed and its degrees of freedom is $m_1$. Let $F(T_\textrm{obs}^2; m_1, \lambda )$ denote the cumulative distribution function (CDF) of the non-central Chi-squared distribution the test statistic $T^2_{(o)}$ follows. Then $F(T_\textrm{obs}^2; m_1, \lambda$) is a monotonic and strictly decreasing function of the NCP, $\lambda$.

The lower bound ($\ell$) and upper bound (*u*) of central $(1 - \alpha )\times 100\%$ CI for $\lambda$ can be chosen as the values that satisfy the equalities (Kent & Hainsworth, 1995)

$$\begin{aligned} F(T_\textrm{obs}^2; m_1, \lambda = \ell ) = 1-\alpha /2\\ F(T_\textrm{obs}^2; m_1, \lambda = u) = \alpha /2 \end{aligned}$$

For the non-central F distribution, the procedure to construct a CI for its NCP is similar, except there are 2 degree of freedom parameters. The lower ($\ell$) and upper (*u*) bounds for the NCP of the F distribution can be determined from the observed statistic, $T_\textrm{obs}^2$, by choosing $\ell$ and *u* to satisfy

$$\begin{aligned} F(T_\textrm{obs}^2; m_1, n-m, \lambda = \ell ) = 1-\alpha /2\\ F(T_\textrm{obs}^2; m_1, n-m, \lambda = u) = \alpha /2, \end{aligned}$$

with degrees of freedom being $m_1$ and $(n-m)$. These are commonly used intervals included in R package MBESS, but the functions in MBESS may return missing values when the test statistic or the sample size is too small. We also provide code to compute these intervals in the RESI R package (https://cran.r-project.org/web/packages/RESI/index.html).Our code is adapted from the code in MBESS, Kent & Hainsworth (1995), and Steiger & Fouladi (1997).

#### Resampling Methods: Using Bootstraps

As we discussed in the previous section, the distribution of the test statistic may deviate from the theoretical non-central Chi-squared or F distribution if the covariance must be estimated and/or the design matrix is random instead of fixed. In this situation, the CIs built through non-central distributions may have lower than nominal coverage of the true effect size. Bootstrapping is a good alternative that can be used to approximate the actual distribution of the test statistic and can be used to construct the intervals for test statistic and the estimated effect size (Preacher & Kelley, 2011). In the body of this paper, we consider the standard nonparametric bootstrap and Bayesian bootstraps (Efron, 1979; Rubin, 1981; Hall, 1992).

*Nonparametric bootstrap:*

We consider the standard nonparametric bootstrap by sampling the data with replacement *R* times and estimating the corresponding RESI for each resampled data. The lower and upper $\alpha /2\times 100\%$ percentiles of the *R* estimated RESIs are the bootstrapped lower and upper bounds of the $(1-\alpha )\times 100\%$ bootstrap CI for the estimated RESI. The nonparametric bootstrap approximates the sampling distribution of the RESI estimator.

*Bayesian bootstrap:*

We also consider a Bayesian bootstrap (Rubin, 1981) as an alternative to the nonparametric bootstrap. The Bayesian bootstrap samples weights for each observation/subject in the data from Dirichlet distribution for *R* times and estimates the corresponding RESI using a weighted regression model for each replicate of the Bayesian bootstraps. The $(1 - \alpha ) \times 100\%$ Bayesian credible interval can be derived using the lower and upper $\alpha /2 \times 100\%$ percentiles of the *R* estimated RESIs from the Bayesian bootstrap replicates.

The Bayesian bootstrap is advantageous when the dataset is not suitable for nonparametric bootstraps. For example, when we have an analysis dataset containing a binary predictor variable which has low variability (say, 98% of the observations are ones). In this case, the values in the binary variable in a bootstrapped dataset may be completely identical and the nonparametric method will fail due to rank deficiency, whereas Bayesian bootstrap is still functional given such analysis data. In addition, the resulting credible intervals provide Bayesian interpretation.

Let *W* denote the dataset and the $g = (g_1, g_2, \dots , g_n)$ denote the sampling weights of the datapoints which follow Dirichlet distribution Dirichlet($1,\dots , 1$), then the posterior distribution of the sampling weights is

$$\begin{aligned} P(g \mid W) = \frac{P(W \mid g) \times P(g )}{P(W)}. \end{aligned}$$

Then the posterior distribution of the RESI parameter is

11 $$\begin{aligned} P(S \mid W )&= \int _g P(S \mid W, g) P(g \mid W) d g \nonumber \\&= \int _g \frac{P(W \mid S, g) P(S) P(g)}{P(W)} d g \end{aligned}$$

where the second line follows by assuming a non-informative improper prior on the effect size *S* (i.e., $P(S) \propto 1$) and the non-informative Dirichlet prior on the sampling weights (Rubin, 1981). Therefore, by conducting the Bayesian bootstrap procedure described above, we simulate the posterior distribution of the RESI in (11).

In addition to nonparametric and Bayesian bootstraps, we also considered several variants of the wild bootstrap (Wu, 1986). However, these wild bootstraps do not show exceeding performance compared to nonparametric and Bayesian bootstraps, and they carry more assumptions. Therefore, we focus on the nonparametric and Bayesian bootstraps in this paper. Details about the wild bootstraps considered in this paper and their simulation results can be found in the Supplementary Material.

## Illustration of Data Analysis Using RESI

In this section, we use two datasets from studies of relational memory among schizophrenia and early psychosis patients (Armstrong et al., 2012; Avery et al., 2021) to illustrate how to report RESI estimates and CIs in an ANOVA-table format as an addition to the conventional model results, using the function resi in the RESI R package. Relational memory is the ability to bind information into complex memories and is impaired in chronic schizophrenia and in the early stages of psychosis (Armstrong et al., 2012; Armstrong et al., 2018; Avery et al., 2021). In both studies a relational memory paradigm was used to compare the ability of psychosis patients and healthy individuals to identify novel stimulus pairings (Armstrong et al., 2012; Armstrong et al., 2018; Avery et al., 2021). The first study compared relational memory accuracy in 60 patients with schizophrenia or schizoaffective disorder to 38 healthy control subjects (Armstrong et al. 2012). The second study assessed participants’ relational memory accuracy in 66 early psychosis patients and 64 healthy control subjects (Avery et al., 2021).

In study reporting, we recommend including RESI and its CI in addition to classic statistical values. The magnitudes of effect on RESI can be described using the thresholds determined by Cohen (1988) (Vandekar et al., 2020), but these are suggestions as interpretations of magnitudes will be field specific. The RESI differs from the *p*-value because it serves to indicate the estimated strength of the effect independent of the sample size. The RESI CI serves to identify the plausible range of values for the effect size. The *p*-value represents how unlikely a result at least as extreme as the observed one is, given the null hypothesis is true. The analyses below serve to illustrate how to report effect sizes along with classical statistical values.

In our analyses, in order to demonstrate the communicability of the RESI across different models, we use both logistic regression models and multiple linear models to quantify the differences in relational memory performance in schizophrenia and early psychosis after controlling for age and gender, respectively (See Appendix for R code). Then, the RESI and corresponding CIs are estimated to indicate the effect size of each factor after controlling others. The results are summarized in an ANOVA table format (see Tables 1 and 2). The RESI estimates and its CIs are based on the model fit object using the resi function in R with the robust covariance estimator, and the 1,000 nonparametric bootstrap samples.

In the first study of patients with schizophrenia, we fit a logistic regression model to study the effect of diagnosis on relational memory compared to healthy controls (Table 1). There is a medium to large association between schizophrenia and relational memory after controlling for age and gender (RESI $=0.44$, 95% CI = [0.25, 0.63]), and there is sufficient statistical evidence to reject the null hypothesis ($\chi ^2 = 18.92$, $d.f. = 1$, $p < 0.001$).

The estimated RESIs and their CIs from logistic and linear models are very close to each other (Table 1). If one model had much greater sensitivity to a given effect, it would be represented by a larger effect size estimate comparing across the models. Using the RESI makes it easier to compare across logistic and linear models.

**Table 1:** A comparison of effect sizes in the schizophrenia (SZ) data from logistic and linear models. The observed RESIs and their CIs for SZ after controlling for age and gender are very close from the two different models, which demonstrates the robustness of RESI across different models. The *p*-values are calculated from the Chi-squared distribution.

**Logistic model: schizophrenia (SZ) vs. healthy controls**							
Factor	Estimate	Robust s.e.	Chi-squared	d.f.	*p*-value	RESI	95% CI
Group (SZ)	–0.77	0.18	18.92	1	<0.001	0.44	(0.25, 0.63)
Age	–0.03	0.01	14.18	1	<0.001	0.37	(0.17, 0.59)
Gender (female)	–0.15	0.14	1.19	1	0.27	0.05	(0, 0.30)
**Overall**			50.55	3	<0.001	0.71	(0.53, 1.02)
Residual				94			
**Linear model: schizophrenia (SZ) vs. healthy controls**							
Group (SZ)	–0.17	0.04	20.99	1	<0.001	0.46	(0.24, 0.69)
Age	–0.01	0.00	15.35	1	<0.001	0.39	(0.18, 0.65)
Gender (female)	–0.03	0.03	1.00	1	0.32	0	(0, 0.29)
**Overall**			72.76	3	<0.001	0.86	(0.60, 1.38)
Residual				94			

In the second study of early psychosis (EP) patients, we fit a logistic regression model to study the effect of psychosis on relational memory compared to healthy controls (Table 2). Results show a medium to large effect of early psychosis on the relational memory after controlling for age and gender (RESI $= 0.49$, 95% CI = [0.31, 0.72]). There is sufficient statistical evidence to reject the null hypothesis ($\chi ^2 = 31.43$, $d.f. = 1$, $p < 0.001$). As above, the logistic and linear models provide similar conclusions (Table 2).

The RESI also makes it easy to compare findings across these two studies that have different sample sizes. The effect sizes are both large and similar in magnitude across the two studies indicating a similar relational memory deficit in EP and SZ. While the test statistics and *p*-values are sample size dependent, the RESI can be compared across the four models that come from two different samples and two model types.

**Table 2:** A comparison of effect sizes in the early psychosis (EP) data from logistic and linear models. The observed RESIs and their CIs for EP after controlling for age and gender are very close from the two different models, which demonstrates the robustness of RESI across different models. The *p*-values are calculated from the Chi-squared distribution.

**Logistic model: early psychosis (EP) vs. healthy controls**							
Factor	Estimate	Robust s.e.	Chi-squared	d.f.	*p*-value	RESI	95% CI
Group (EP)	–1.26	0.22	31.43	1	<0.001	0.49	(0.31, 0.72)
Age	0.04	0.04	0.79	1	0.37	0	(0, 0.28)
Gender (female)	0.33	0.27	1.50	1	0.22	0.06	(0, 0.26)
**Overall**			45.17	3	<0.001	0.58	(0.43, 0.82)
Residual				126			
**Linear model: early psychosis (EP) vs. healthy controls**							
Group (EP)	–0.18	0.03	32.54	1	<0.001	0.50	(0.32, 0.71)
Age	0.01	0.01	0.93	1	0.33	0	(0, 0.29)
Gender (female)	0.04	0.03	1.73	1	0.19	0.08	(0, 0.29)
**Overall**			45.59	3	<0.001	0.58	(0.44, 0.82)
Residual				126			

## Simulation Study

### Setup

In the previous sections, we proposed three estimators and several ways of constructing (confidence/credible) intervals for the RESI. In this section, we use 1,000 simulations to evaluate the performance of the proposed interval construction procedures with respect to each estimator under different scenarios. We evaluate the influence of heteroskedasticity, data skewness and fixed/random covariate(s) on the performance of estimators and intervals in some particular situations that we believe represent reasonable conditions researchers care about in practice. All intervals are constructed at the 95% confidence/credible level.

We simulate a simple linear regression model $Y = \beta _0 + \beta _1 x + \epsilon$, where $x \in \{0, 1\}$. We vary the sample size $n \in \{ 50, 100, 250, 500\}$ across 4 different true effect sizes, $S \in \{0, 0.33, 0.66, 1\}$. In the scenario of symmetric errors, the errors are independently sampled from $N(0, \sigma _0^2 = 1)$ under homoskedasticity; under heteroskedasticity, the errors are independently sampled from $N(0, \sigma _0^2 = 0.25)$ if $x_i = 0$ and from $N(0, \sigma _1^2 = 2.25)$ if $x_i = 1$. In the scenario of skewed errors, $\epsilon + \sqrt{0.1}$ $\sim$ Gamma($\alpha = 0.1$, $\beta = \sqrt{0.1}$) under homoskedasticity and $\epsilon + \sqrt{0.1} (x+0.5)$ $\sim$ Gamma($\alpha = 0.1$, $\beta = \sqrt{0.1}/(x+0.5)$) under heteroskedasticity. These errors are very heavily right-skewed, with Pearson’s moment coefficient of skewness equal to $\frac{mean - mode}{\sqrt{Variance}} = \left( \frac{\alpha }{\beta } - \frac{\alpha - 1}{\beta } \right) /\sqrt{\alpha /\beta ^2} = 2/\sqrt{0.1} \approx 6.32$. For a symmetric distribution, this skewness coefficient is 0. The errors are shifted so that they are mean zero.

To illustrate the difference in the performance of the intervals between an experimental and observational design where the covariate(s) *X* is treated as fixed or random, the values of the covariate were generated in two ways: (1) when the covariate is fixed, $\pi = 0.3$ and ceiling($n\pi$) of the *n* individuals have their covariate with value 1 and the remaining have value 0; (2) when the covariate is random, $X \sim \textrm{Bern}(0.3)$ is sampled from a Bernoulli distribution with parameter $\pi = 0.3$. For each bootstrap intervals, 1,000 bootstraps are used.

### Results

We ran simulations to assess the bias of the estimators and the coverage of the confidence/credible intervals. We considered eight possible cases where there are homo- or heteroskedastic errors, symmetric or skewed errors, and fixed or random covariates in each simulation.

**Fig. 2:**
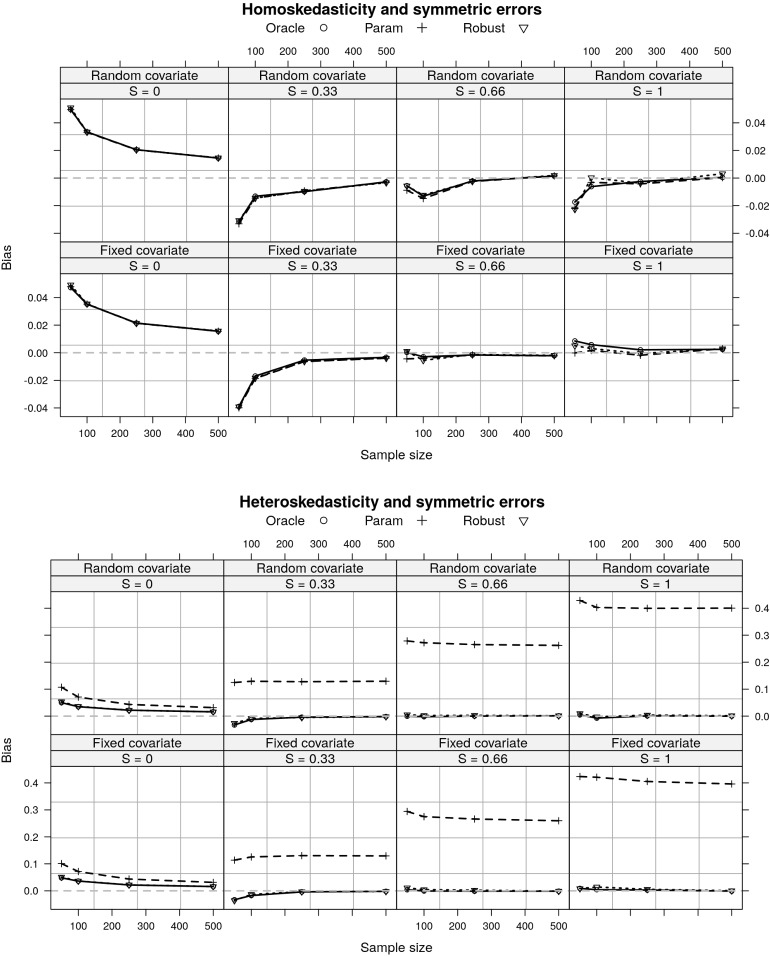
A comparison of bias for three estimators of the RESI under the simulation settings described in Sect. 4. Under homoskedasticity the estimators have small bias (top). Under heteroskedasticity, the parametric estimator for effect size is heavily biased. *S* denotes the RESI, Oracle estimator assumes known variance, parametric assumes homoskedasticity, Robust is the sandwich covariance estimator (Sect. 1.2).

**Fig. 3:**
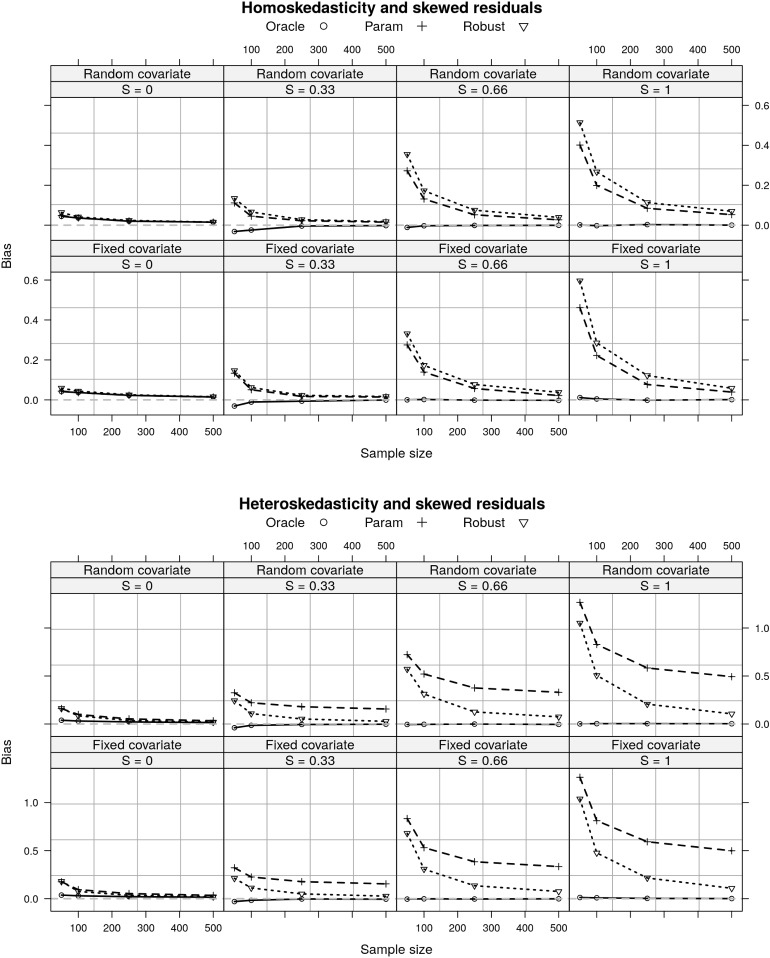
A comparison of bias for three estimators of the RESI under the simulation settings described in Sect. 4. Under homoskedasticity and heavily skewed residuals, the estimators have bias (top), but the bias goes to 0 in large samples. Under heteroskedasticity and heavily skewed residuals, the parametric estimator for effect size is heavily biased, the bias of the robust estimator approaches 0 in large samples. *S* denotes the RESI, Oracle estimator assumes known variance, parametric assumes homoskedasticity, Robust is the sandwich covariance estimator. (Sect. 1.2).

In small samples ($n=50$) the estimators are positively biased for $S=0$, but negatively biased for all other values of *S* (Fig. 2). For all other sample sizes, ${\hat{S}}$ has small bias. As expected, under heteroskedasticity, the parametric estimator is heavily biased (Vandekar et al., 2020). When the error distribution is heavily skewed and $S>0$, the robust estimator is biased, but the bias goes to zero in large samples (Fig. 3). The randomness of the covariate does not have an effect on the consistency of any of these 3 estimators.

**Fig. 4:**
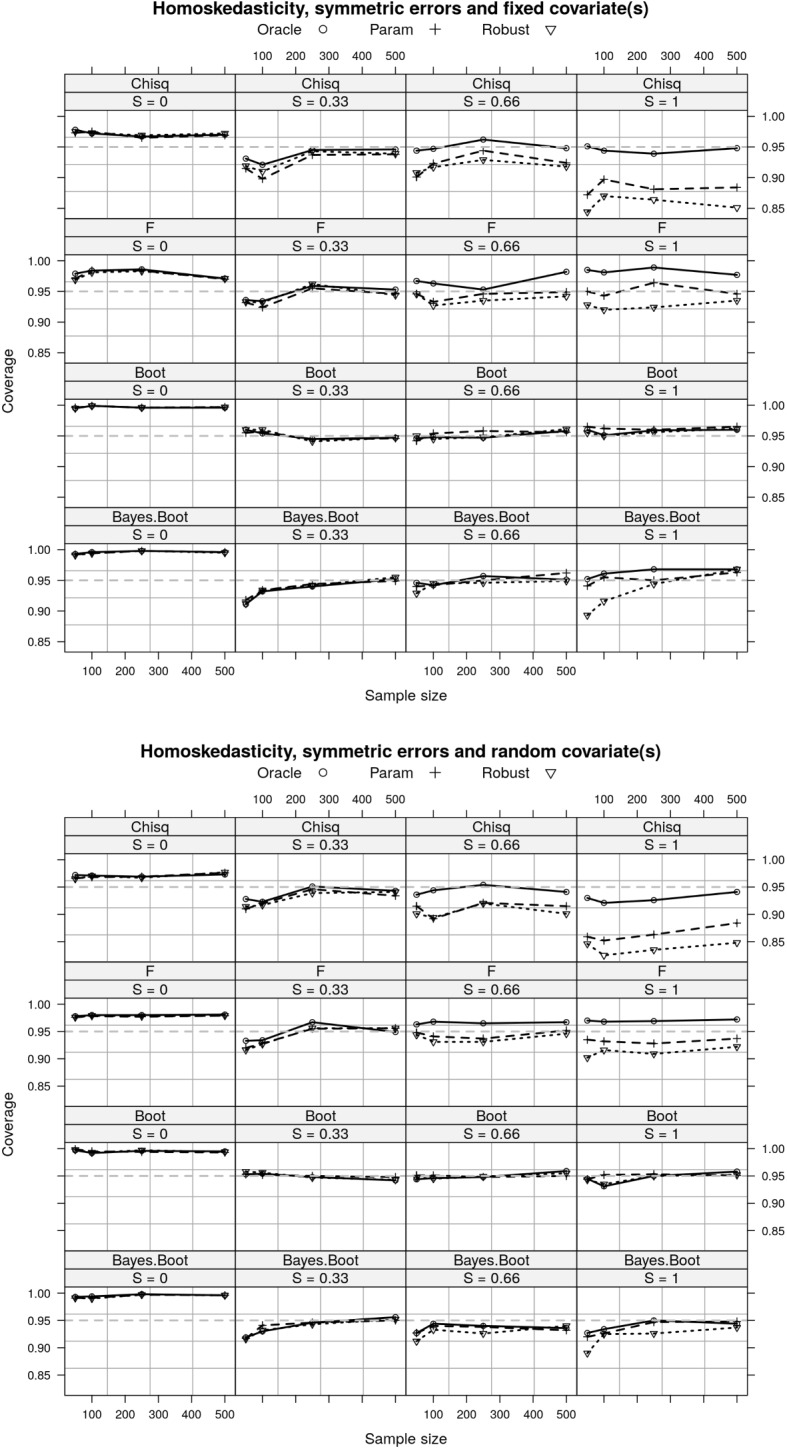
A comparison of the coverage of different CIs for the three estimators of RESI under homoskedasticity, with symmetric residuals and random/fixed covariates. With fixed covariates, the Chi-squared CI has nominal coverage for the oracle estimator and F CI has nominal coverage for the parametric estimator, whereas they both fail when the covariates are random. The nonparametric bootstrap CI has nominal coverage for all estimators, with random or fixed covariates. The Bayesian interval has similar performance with the nonparametric bootstrap CI for the robust estimator. *S* denotes the RESI; Oracle estimator assumes known variance; parametric assumes homoskedasticity; Robust is the sandwich covariance estimator (Sect. 1.2); Chisq denotes Chi-squared CI; F denotes F CI; Boot denotes nonparametric bootstrap CI; Bayes Boot denotes the Bayesian interval.

As to the coverage of each CI approach, the pattern of differences between the oracle, parametric, and robust estimators is most obvious when the effect size is larger (e.g., $S=1$). In this case of homoskedasticity, symmetric errors and fixed covariate (the upper plot in Fig. 4), the Chi-squared CI has nominal coverage when we have known covariance (i.e., the oracle estimator), the F CI has nominal coverage when we use naive covariance estimator (i.e., the parametric estimator) as expected, and the nonparametric bootstrap CI has nominal coverage for all (asymptotically). The Bayesian interval has comparable performance as the nonparametric bootstrap CI, but performs slightly worse when the sample size is very small. As expected, larger effect sizes have worse coverage for the Chi-squared and F CIs when the variance is estimated (parametric and robust statistics; Fig. 4) because the variance depends on the true effect size and sample size, and the asymptotic distributions don’t hold. This is as expected from the results in Fig. 1 that the asymptotic variance of all three test statistics are not equal. The variance of the robust estimator is asymptotically larger than the variance of the parametric estimator, which in turn is asymptotically larger variance than the oracle estimator. The relation between the variance of the parametric and oracle estimators is given in equation (9).

When there is a random covariate, the Chi-squared and F CIs both fail to provide nominal coverage for the oracle and parametric estimators, respectively. This is because the extra variance introduced by random covariate into the test statistics and the asymptotic distribution do not hold any more (also shown in Fig. 1).

**Fig. 5:**
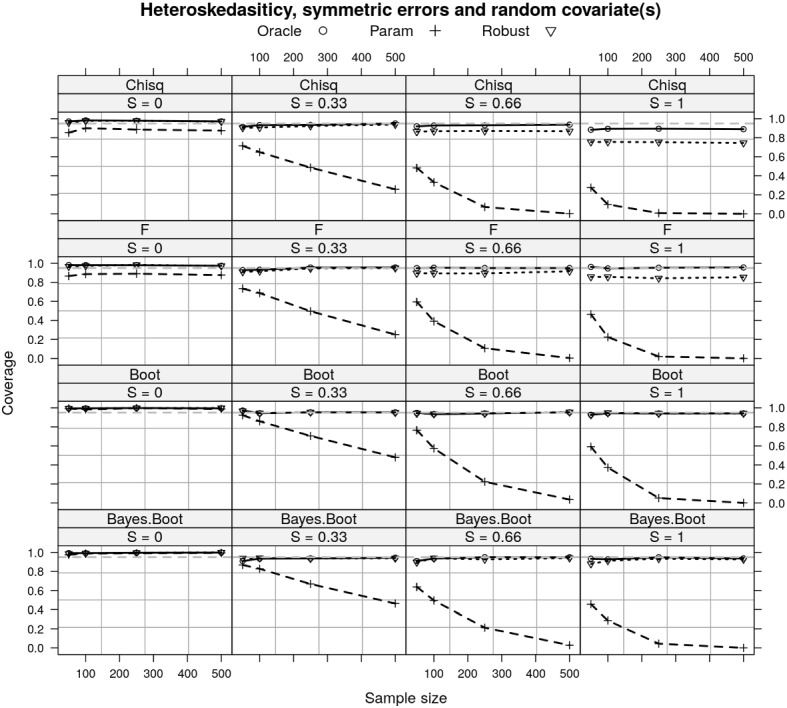
A comparison of the coverage of different intervals for the three estimators of RESI under heteroskedasticity, with symmetric residuals and random covariates. All three CIs fail to provide nominal coverage for the parametric estimator. The Chi-squared and F CIs fail to provide nominal coverage for the robust estimator. The nonparametric bootstrap CI and Bayesian interval has nominal coverage for both the oracle and robust estimators. *S* denotes the RESI; Oracle estimator assumes known variance; parametric assumes homoskedasticity; Robust is the sandwich covariance estimator (Sect. 1.2); Chisq denotes Chi-squared CI; F denotes F CI; Boot denotes nonparametric bootstrap CI; Bayes Boot denotes the Bayesian Interval.

Under heteroskedasticity, all intervals fail to provide nominal coverage using the parametric estimator because this estimator is biased as shown in Fig. 2. Both Chi-squared and F CIs fail to provide nominal coverage for the robust estimator. The nonparametric bootstrap CI and Bayesian interval have nominal coverage for both of the oracle and robust estimators (Fig. 5).

**Fig. 6:**
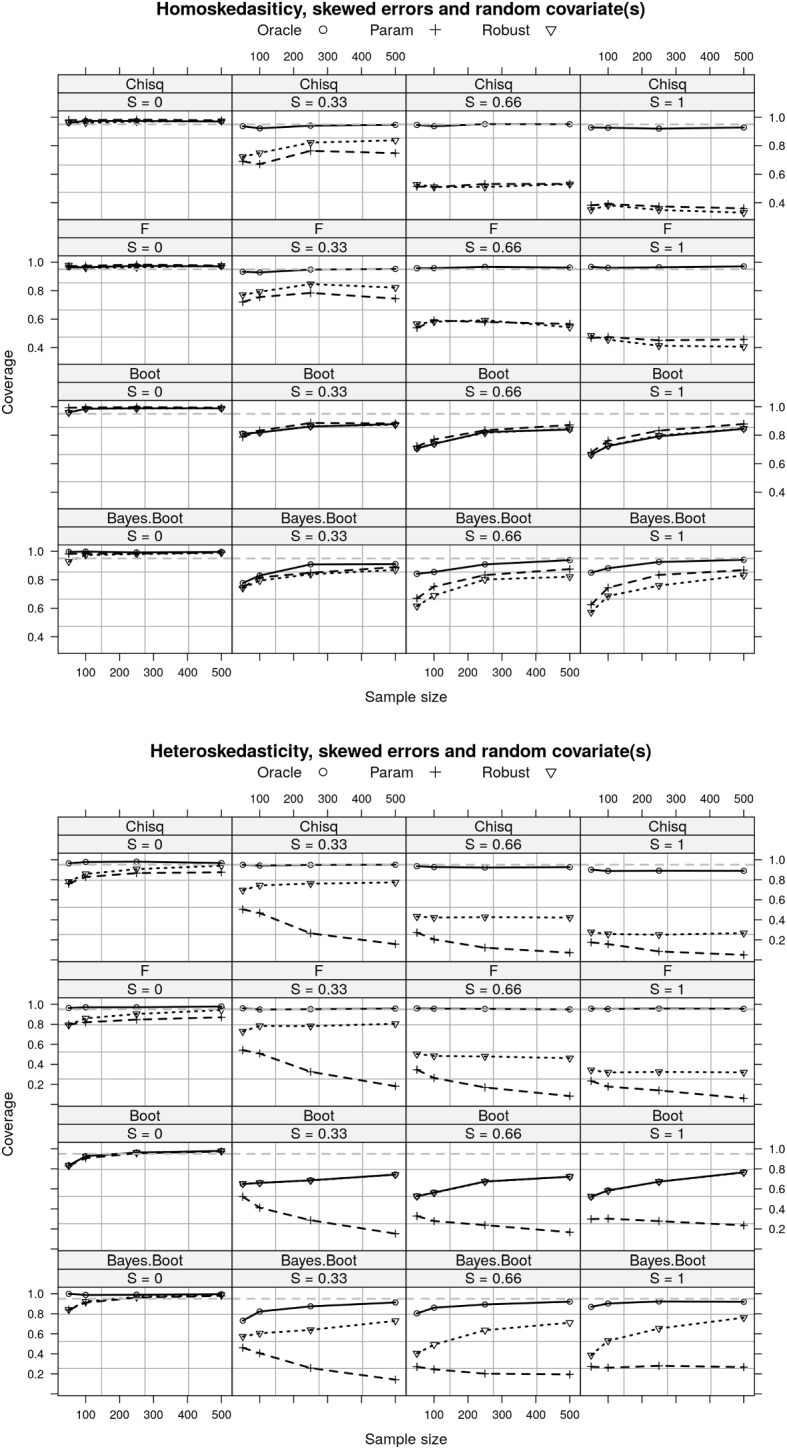
A comparison of the coverage of different CIs for the three estimators of RESI with skewed residuals and random covariates. The coverages of the nonparametric bootstrap CI and Bayesian interval approach to the nominal level in large sample for the oracle and robust estimators in both homo- and hetero-skedasticity. *S* denotes the RESI; Oracle estimator assumes known variance; parametric assumes homoskedasticity; Robust is the sandwich covariance estimator (Sect. 1.2); Chisq denotes Chi-squared CI; F denotes F CI; Boot denotes nonparametric bootstrap CI; Bayes Boot denotes the Bayesian Interval.

The skewness of the residuals has a big impact on the intervals’ performance. Under homoskedasticity, the Chi-squared and F CIs provide nominal coverage for the oracle estimator, but they both fail to provide nominal coverage for the parametric and robust estimators (Fig. 6). In large samples, the coverage of the nonparametric bootstrap CI and the Bayesian interval for all three estimators approach the nominal level. Under heteroskedasticity, all intervals fail to provide nominal coverage for all estimators except F CI for the oracle estimator. The coverage of the nonparametric bootstrap CI and the Bayesian interval for the oracle and robust estimators approaches the nominal level in large samples.

## Discussion

In this paper, we derived confidence and credible intervals for the robust effect size index (RESI) and used them to to easily report effect sizes and their CIs in an ANOVA table format. We proposed 3 different estimators for the RESI and a variety of ways to construct intervals. The oracle estimator assumes the covariance matrix is known, so is not possible to compute in applications. Through simulations, we showed that all 3 estimators are consistent under homoskedasticity and the robust estimator is consistent under heteroskedasticity. We used statistical theory and simulations to demonstrate that the non-central Chi-squared CI has low coverage when the covariance in the Wald test statistic needs to be estimated. In addition, the randomness of covariate(s) also reduces the coverage of Chi-squared and F CIs, which is an important implication for observational studies where the covariates are random instead of fixed/controlled by the experimenters. According to the simulation results, using the robust estimator along with the (nonparametric) bootstrap CI is generally most accurate and applicable to conduct consistent estimation and valid inference using the RESI. The credible interval has comparable performance to the nonparametric bootstrap CI for the robust estimator. It also has a Bayesian interpretation and can be used as an alternative to the nonparametric bootstrap CI.

The RESI estimator and CI reduce the barriers to effect size analysis by introducing a unified reporting procedure that is widely applicable across different models. The effect sizes with confidence or credible intervals can easily be reported in summary tables alongside *p*-values. This approach may help to address the limitations of null hypothesis significance testing (Wasserstein & Lazar, 2016; Wasserstein et al., 2019) and provide guidance on whether a study finding is under-powered. We hope the ease of using RESI will broaden the use of effect sizes and their CIs in study reporting.

We also provided functions to estimate RESI and its CI in our RESI R package (https://cran.r-project.org/web/packages/RESI/index.html). It outputs the estimated RESI along with intervals in a summary table, with which researchers can conveniently report their observed results. Coupled with the generality of the RESI, researchers studying the same scientific questions but using different types of data can easily communicate their observed effect sizes and confidence/credible intervals instead of having to translate between different effect size indices.

Our research focused on the performance of the confidence/credible intervals in a linear regression model setting because these are widely used models. Although it adequately illustrated the problems we wanted to discuss, theory and evaluation for other models (e.g., longitudinal and survival analysis) requires further research.

### Supplementary Information

Below is the link to the electronic supplementary material.

Supplementary file 1 (pdf 1557 KB)

## Appendix

### R Code

In this section, we illustrate how to analyze and report RESI estimates and their CIs in the relational memory datasets using the functions from the RESI R package, and the complete code for our data analyses are also included. The two datasets are described in section 3 and have already been loaded into R. Each of these two datasets contains 4 variables “accuracy” (the outcome), “group”, “age” and “gender”. We first fit a glm object by specifying the variable accuracy as the outcome, it can be a logistic regression model if we specify family = “binomial” or a linear model if family = “gaussian” (default). Note, for both models, the assumptions are violated because, for the logistic regression the data are not Bernoulli distributed (they are proportions) and for the Gaussian model, the errors are not normal. Then the RESI estimates and CIs can be calculated with resi(object = glm.fit), where glm.fit is the glm object we just created.

The complete codes for our analyses are:

By default, resi uses the robust (sandwich) estimator (robust.var = TRUE, default) to estimate the RESI, if robust.var = FALSE, it uses (4) to calculate the parametric test statistics (7) and corresponding parametric version of RESI estimates. resi uses 1,000 bootstraps (nboot = 1000) to construct the corresponding intervals. The default interval construction method is nonparametric bootstrapping (boot.method = “nonparam”), Bayesian bootstrapping can be implemented instead by setting boot.method = “bayes”. alpha is the significance level at which the intervals are estimated, by default, alpha = 0.05;

If we are interested in estimating the overall effect size of a subset of factors (e.g., age and gender in our example), we can specify a reduced model to compare with (e.g., $\mathtt{model.reduced = glm(accuracy \sim group, data = data, family = ``binomial'')}$). By default, model.reduced = NULL. The function will then output a table containing the overall effect size of age and gender after controlling for the group. Here is an example:  where the RESI on the “tested” row is the RESI estimate for age and gender together after controlling for group.

### Mathematical Details

To show (8): the distribution is a non-central F distribution.

Under the homoskedasticity and Normality assumption, we have $Y \sim N(X\beta , \sigma ^2 \textbf{I})$.

$$\begin{aligned} T_{(p)}^2 / m_1&= n {\hat{\sigma }}^{-2} ({\hat{\beta }}_{OLS} - \beta _0)^T X^T X ({\hat{\beta }}_{OLS} - \beta _0) / m_1 \\&= \frac{ \sigma ^{-2} n({\hat{\beta }}_{OLS} - \beta _0)^T X^T X ({\hat{\beta }}_{OLS} - \beta _0) / m_1 }{\sigma ^{-2} \hat{\sigma ^2}} \\&= \frac{\sigma ^{-2} n({\hat{\beta }}_{OLS} - \beta _0)^T X^T X ({\hat{\beta }}_{OLS} - \beta _0)}{\sigma ^{-2} Y^T(I - H)Y / (n - m) } \end{aligned}$$

1. In the numerator, under mild conditions, the quadratic form $\sigma ^{-2} n({\hat{\beta }}_{OLS} - \beta _0)^T X^T X ({\hat{\beta }}_{OLS} - \beta _0) \sim \chi ^2(m_1; \lambda _1)$, where $\lambda _1$ is the non-centrality parameter:
  $$\begin{aligned} \lambda _1&= \sigma ^{-2} n(\mathbb {E}{\hat{\beta }}_{OLS} - \beta _0)^T X^T X (\mathbb {E}{\hat{\beta }}_{OLS} - \beta _0) \\&= \sigma ^{-2} n(\beta - \beta _0)^T X^T X (\beta - \beta _0) \\&= nS_{\beta }^2 \end{aligned}$$
  This quadratic form in the numerator is essentially the regression sum of squares (SSR) in linear regression models.
2. In the denominator, the quadratic from $\sigma ^{-2} Y^T(I - H)Y \sim \chi ^2(n-m; \lambda _2)$, where $\lambda _2$ is the non-centrality parameter. It can be shown that $\lambda _2$ is 0:
  $$\begin{aligned} \lambda _2&= \sigma ^{-2} \left[ (X\beta )^T (I - H) X\beta \right] \\&= \sigma ^{-2} \left[ \beta ^T X^T X\beta - \beta ^T X^T H X\beta \right] \\&= \sigma ^{-2} \left[ \beta ^T X^T X\beta - \beta ^T X^T X (X^T X)^{-1} X^T X\beta \right] \\&= \sigma ^{-2} \left[ \beta ^T X^T X\beta - \beta ^T X^T X \beta \right] \\&= 0 \end{aligned}$$
  Therefore, in the denominator, $\sigma ^{-2}Y^T(I - H)Y$ follows a central Chi-squared distribution $\chi ^2_{n-m}$. This quadratic form is essentially the error sum of squares (SSE) in linear regression models.
3. Under homoskedasticity and Normality, the SSR and SSE are independent. Therefore, we can prove that the distribution of $T_{(p)}^2 / m_1$ is the non-central F distribution $F(m_1, n-m; nS^2_{\beta })$
