# Supplementary material for: Accurate Confidence and Bayesian Interval Estimation for Non-centrality Parameters and Effect Size Indices
Source: Psychometrika. 2023 Feb 1;88(1):253–73. doi: 10.1007/s11336-022-09899-x (PMC9977896; doi:10.1007/s11336-022-09899-x)
Supplement: Supplementary file 1 — (pdf 1557 KB) [file 11336_2022_9899_MOESM1_ESM.pdf]

Title: Supplement for *Accurate confidence and Bayesian interval estimation for non-centrality parameters and effect size indices*

Wild Bootstraps

In addition to the non-parametric and Bayesian bootstrap we discussed in the main paper, we also considered several variants of the wild bootstrap, which was originally proposed by (Wu, 1986). The wild bootstrap is a kind of residual bootstrap which leaves the covariates at the sample value but resamples the outcome values based on the residual values. That is, in each replicate, the resampled outcome value of the i-th observation is

$$y_i^* = \hat{y}_i + \hat{\epsilon}_i \times w_i, \quad (1)$$

where $\hat{y}_i$ and $\hat{\epsilon}_i$ are the expected value and estimated residual of the i-th observation from the analysis model, respectively. w_i is a random number, called multiplier, drawn from a distribution W such that $\mathbb{E}(W) = 0$ and $Var(W) = 1$. There are several different distributions from which w_i can be drawn, such as the Rademacher and standard Normal distributions.

The original wild bootstrap doesn't resample the data. We propose several variants of the original wild bootstrap: (1) resampling covariates along with residuals with replacement; (2) fixing covariates and only resampling residuals with replacement; (3) independently resampling covariates and residuals with replacement. We also considered 3 types of multipliers: (1) no multipliers, i.e., $w_i = 1, \forall i$; (2) multipliers drawn from the Rademacher distribution; (3) multipliers drawn from standard Normal distribution. Table 1 below summarizes the original and variant wild bootstraps considered in this paper. The original wild bootstrap without using multipliers is excluded because no resampling happens within this combination. Therefore, there are 11 combinations of the wild bootstrapping evaluated in the simulations.

Wild bootstrap type	Multiplier	Bootstrap model	Assumption(s)
Original	None	$Y^{(b)} = X\hat{\beta} + \hat{\epsilon} \times 1 = Y$	Excluded b/c no sampling happens here
	Rademacher	$Y^{(b)} = X\hat{\beta} + \hat{\epsilon} \times W, w_i \sim Rad$	Symmetric errors
	$N(0,1)$	$Y^{(b)} = X\hat{\beta} + \hat{\epsilon} \times W, w_i \sim N(0,1)$	Symmetric errors
(1) Resampling covariates along with residuals	None	$Y^{(b)} = R^{(b)}X\hat{\beta} + R^{(b)}\hat{\epsilon} \times 1$	Non-parametric bootstrap. not assuming homo-/hetero-skedasticity or symmetric errors
	Rademacher	$Y^{(b)} = R^{(b)}X\hat{\beta} + R^{(b)}\hat{\epsilon} \times W, w_i \sim Rad$	Symmetric errors
	$N(0,1)$	$Y^{(b)} = R^{(b)}X\hat{\beta} + R^{(b)}\hat{\epsilon} \times W, w_i \sim N(0,1)$	Symmetric errors
(2) Fixing covariates and only resampling residuals	None	$Y^{(b)} = X\hat{\beta} + R^{(b)}\hat{\epsilon} \times 1$	Homoskedasticity
	Rademacher	$Y^{(b)} = X\hat{\beta} + R^{(b)}\hat{\epsilon} \times W, w_i \sim Rad$	Homoskedasticity and symmetric errors
	$N(0,1)$	$Y^{(b)} = X\hat{\beta} + R^{(b)}\hat{\epsilon} \times W, w_i \sim N(0,1)$	Homoskedasticity and symmetric errors
(3) independently resampling covariates and residuals	None	$Y^{(b)} = R_1^{(b)}X\hat{\beta} + R_2^{(b)}\hat{\epsilon} \times 1$	Homoskedasticity
	Rademacher	$Y^{(b)} = R_1^{(b)}X\hat{\beta} + R_2^{(b)}\hat{\epsilon} \times W, w_i \sim Rad$	Homoskedasticity and symmetric errors
	$N(0,1)$	$Y^{(b)} = R_1^{(b)}X\hat{\beta} + R_2^{(b)}\hat{\epsilon} \times W, w_i \sim N(0,1)$	Homoskedasticity and symmetric errors

Table 1: Eleven bootstrap procedures considered in this paper. The original wild bootstrap with constant multiplier is excluded since it performs no resampling. $R^{(b)}$ denotes the bootstrap matrix for the b-th replicate; $Y^{(b)}$ is the bootstrapped outcome values; X is the covariate(s); $\hat{\beta}$ is the estimated parameters; $\hat{\epsilon}$ is the estimated residuals; W is the randomly drawn values for the multiplier.

Simulation Results

In this section, we show the coverage performance for the confidence intervals constructed using different wild bootstraps under different scenarios (homo-/hetero-skedasticity and symmetric skewed errors) given *random* covariate and different true RESI value.

Homoskedasticity & Symmetric Errors

$S = 0$

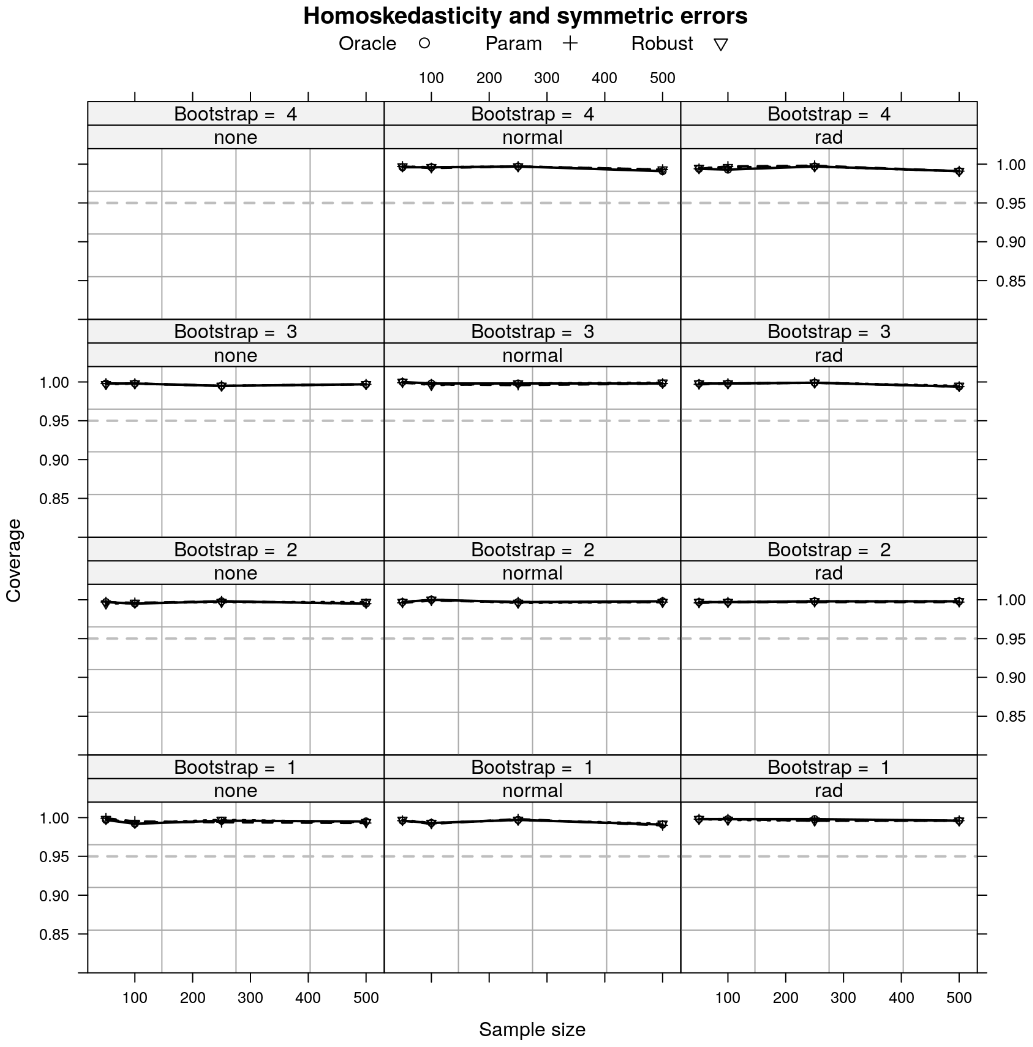

$S = 0.33$

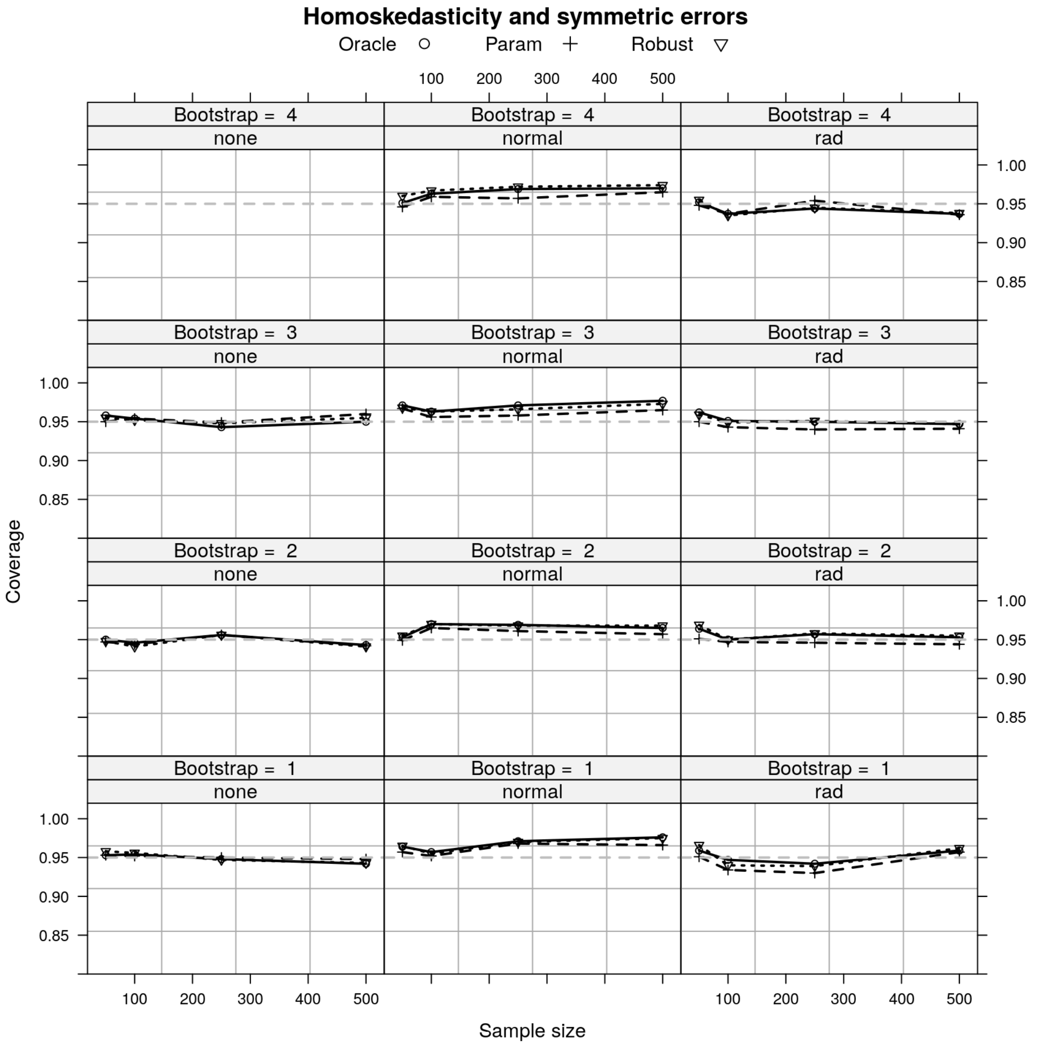
Homoskedasticity and symmetric errors
Oracle
Param
Robust
Bootstrap = 4
Bootstrap = 3
Bootstrap = 2
Bootstrap = 1
none
normal
rad
Coverage
Sample size
100
200
300
400
500
0.85
0.90
0.95
1.00

$S = 0.66$

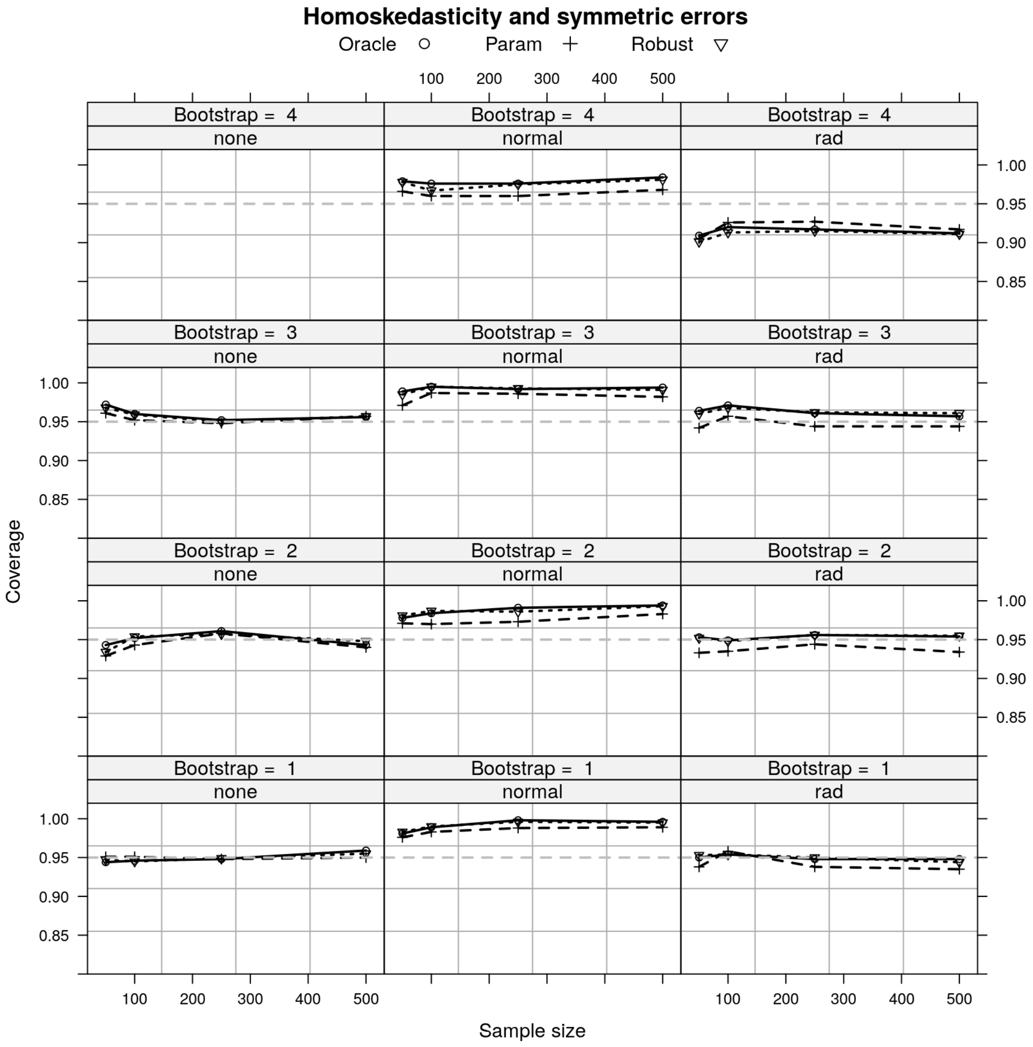

Homoskedasticity and symmetric errors
Oracle
Param
Robust
Bootstrap = 4
Bootstrap = 3
Bootstrap = 2
Bootstrap = 1
none
normal
rad
Coverage
Sample size
100
200
300
400
500
0.85
0.90
0.95
1.00

$S = 1$

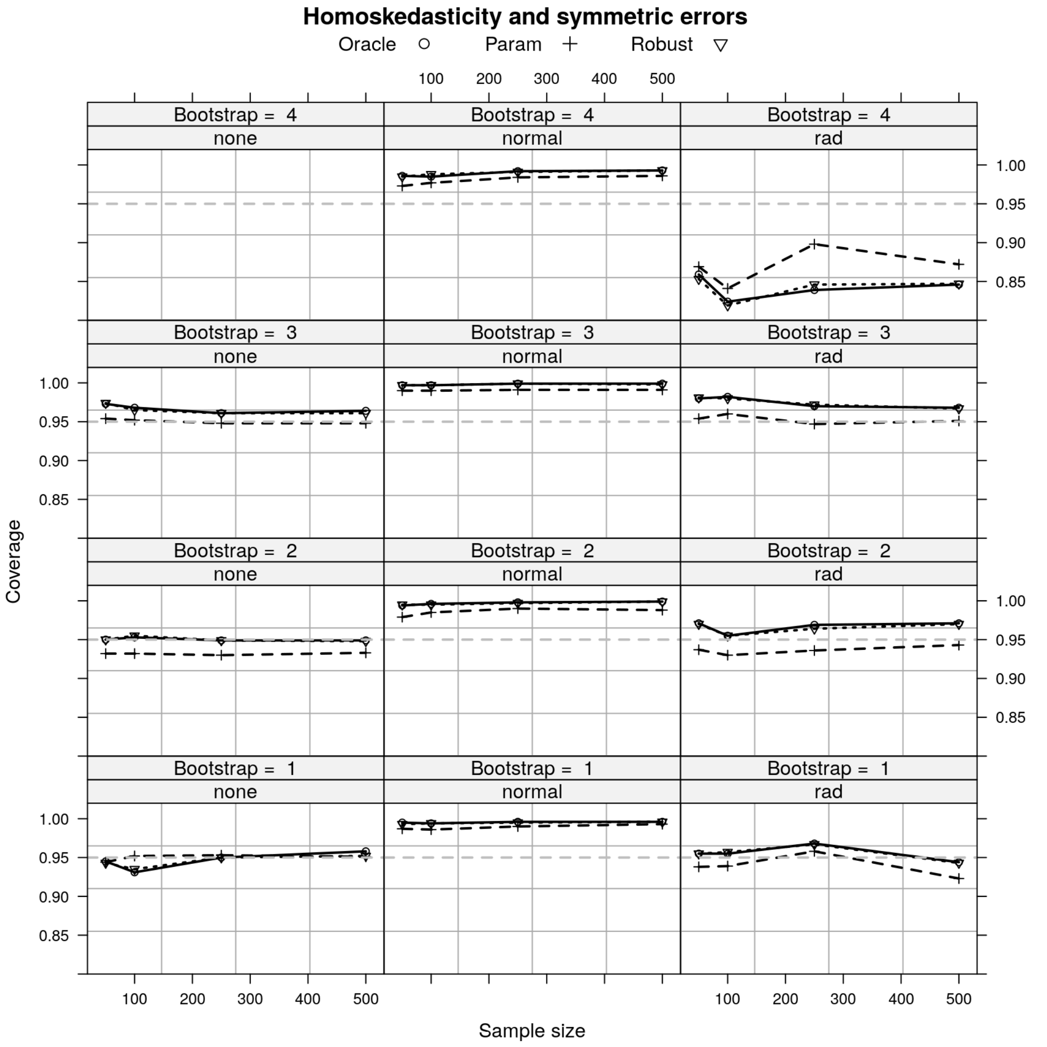
Homoskedasticity and symmetric errors
Oracle
Param
Robust
Bootstrap = 4
none
normal
rad
Bootstrap = 3
Bootstrap = 2
Bootstrap = 1
Coverage
Sample size
100
200
300
400
500
0.85
0.90
0.95
1.00

Heteroskedasticity & Symmetric Errors

$S = 0$

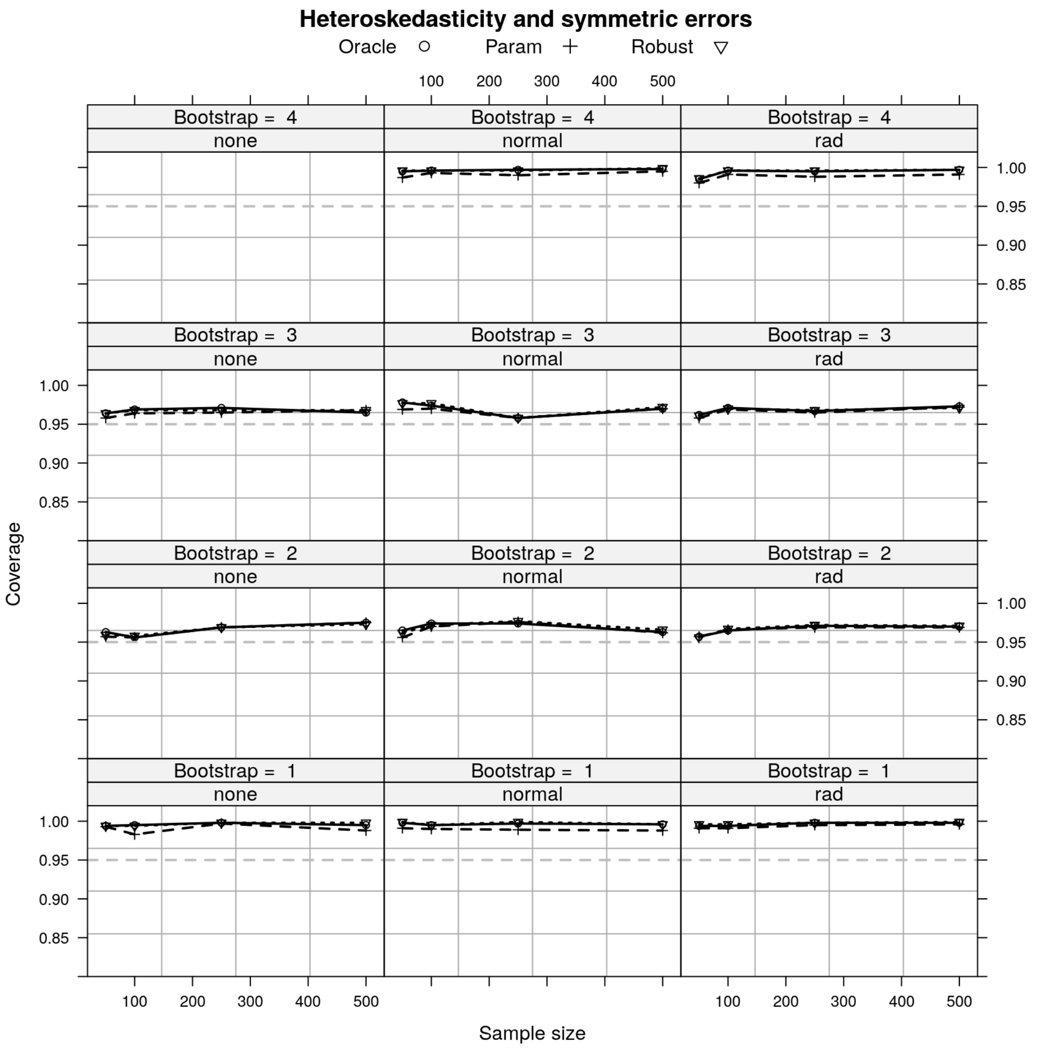

$S = 0.33$

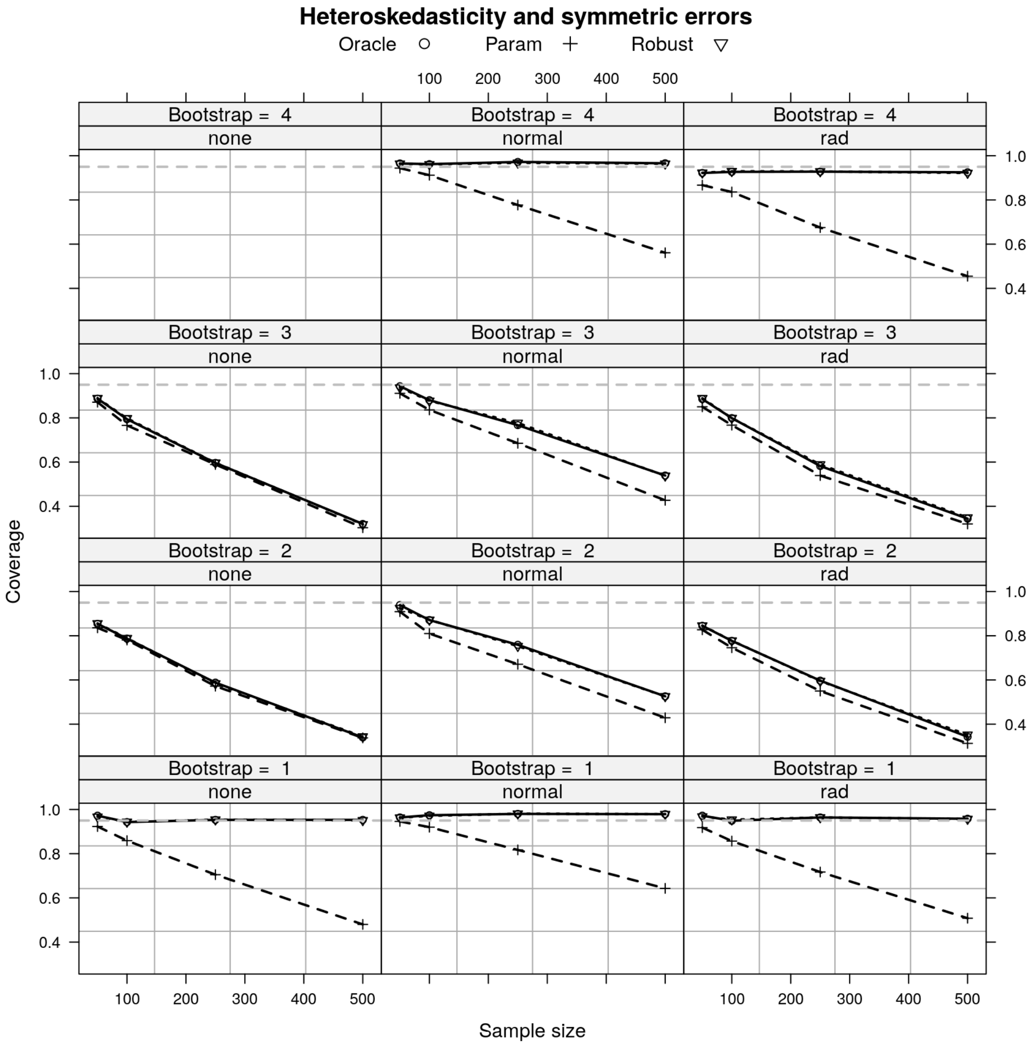
Heteroskedasticity and symmetric errors
Oracle
Param
Robust
Bootstrap = 4
Bootstrap = 3
Bootstrap = 2
Bootstrap = 1
none
normal
rad
Coverage
Sample size
100
200
300
400
500
0.4
0.6
0.8
1.0

$S = 0.66$

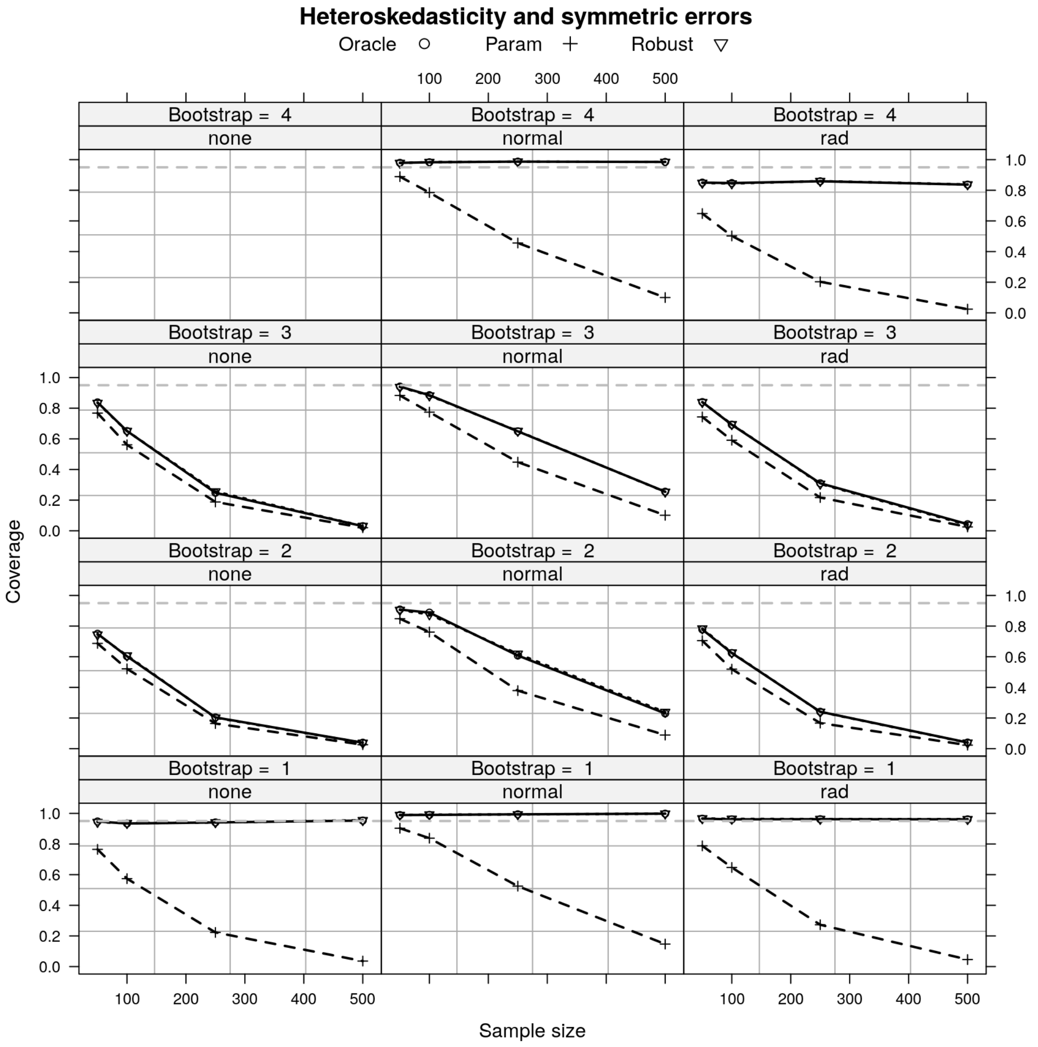

Heteroskedasticity and symmetric errors
Oracle
Param
Robust
Bootstrap = 4
Bootstrap = 3
Bootstrap = 2
Bootstrap = 1
none
normal
rad
Coverage
Sample size
100 200 300 400 500
0.0 0.2 0.4 0.6 0.8 1.0

$S = 1$

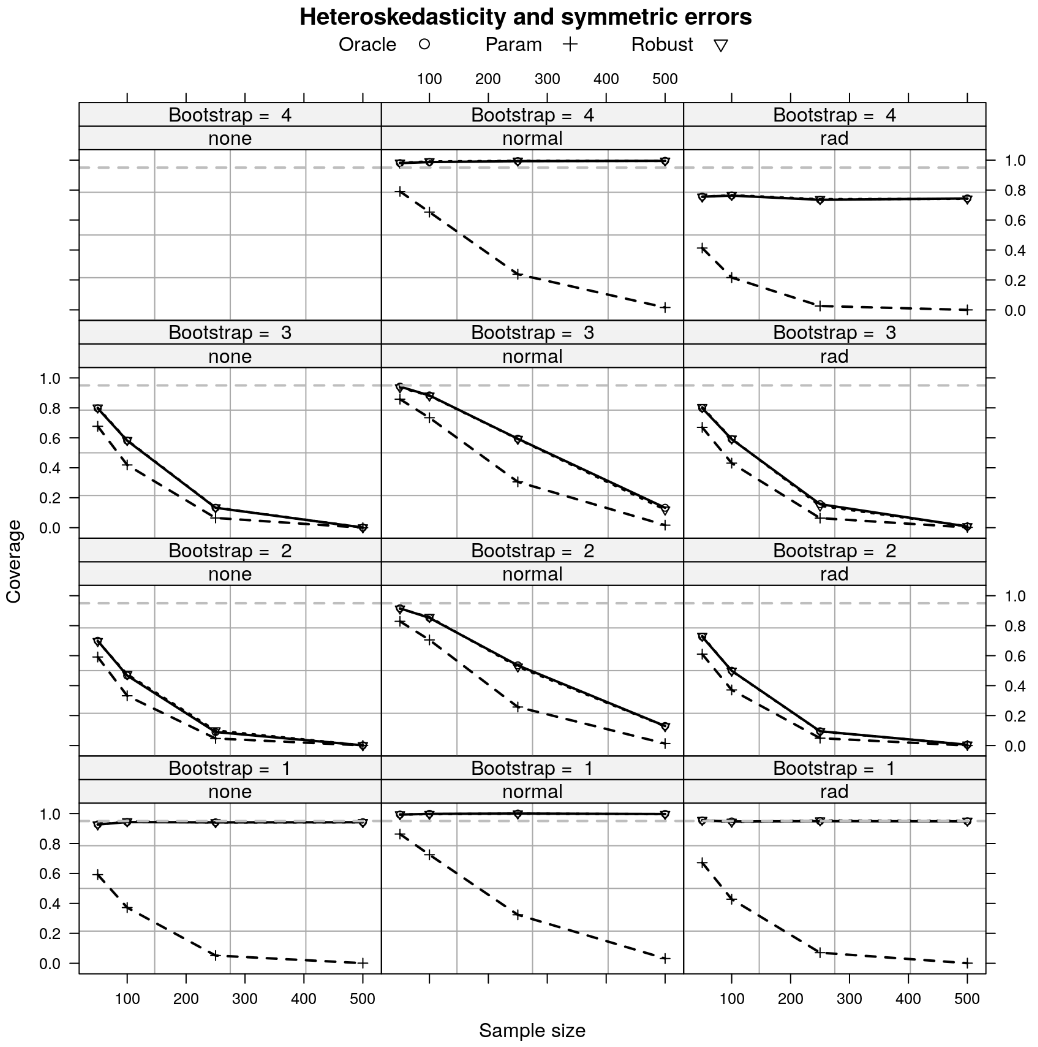

Heteroskedasticity and symmetric errors
Oracle
Param
Robust
Bootstrap = 4
Bootstrap = 3
Bootstrap = 2
Bootstrap = 1
none
normal
rad
Coverage
Sample size
0.0
0.2
0.4
0.6
0.8
1.0
100
200
300
400
500

Homoskedasticity & Skewed Errors

$S = 0$

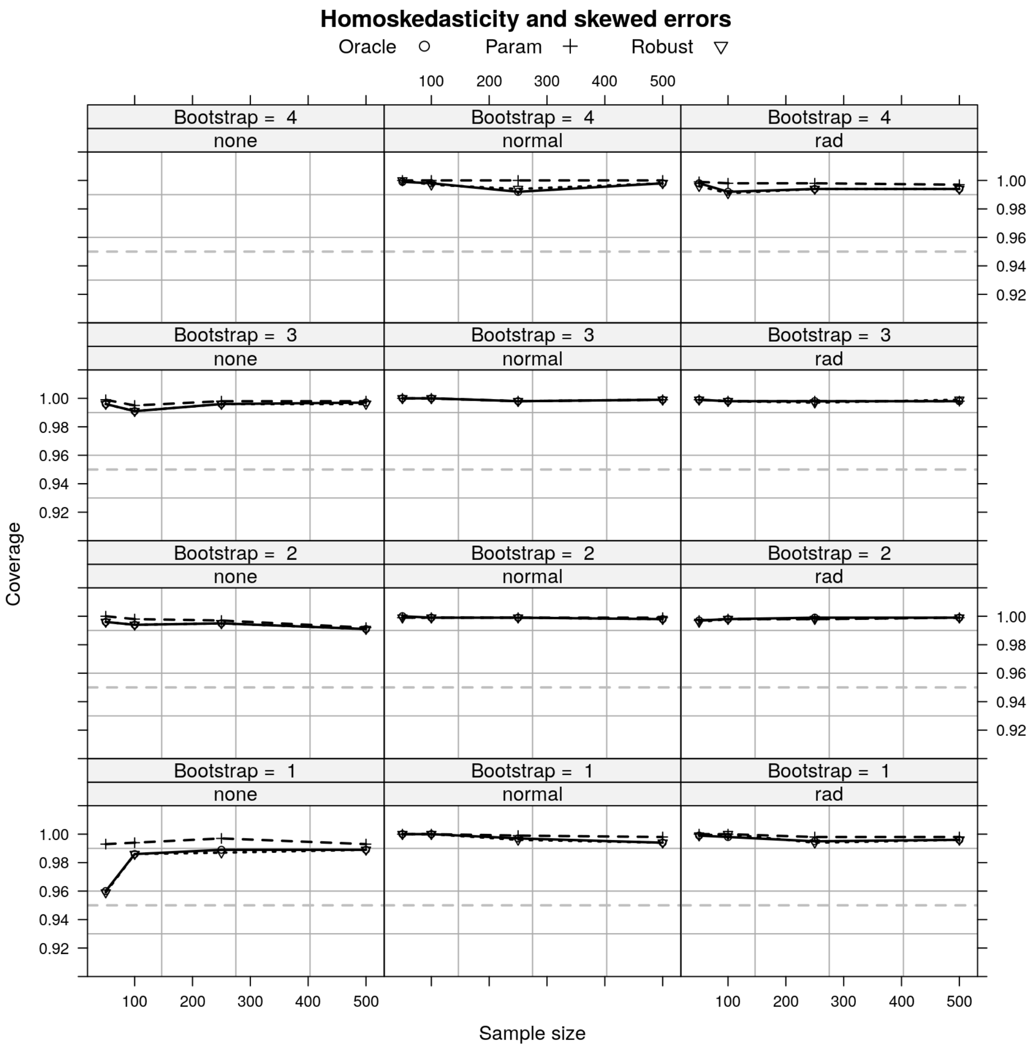

$S = 0.33$

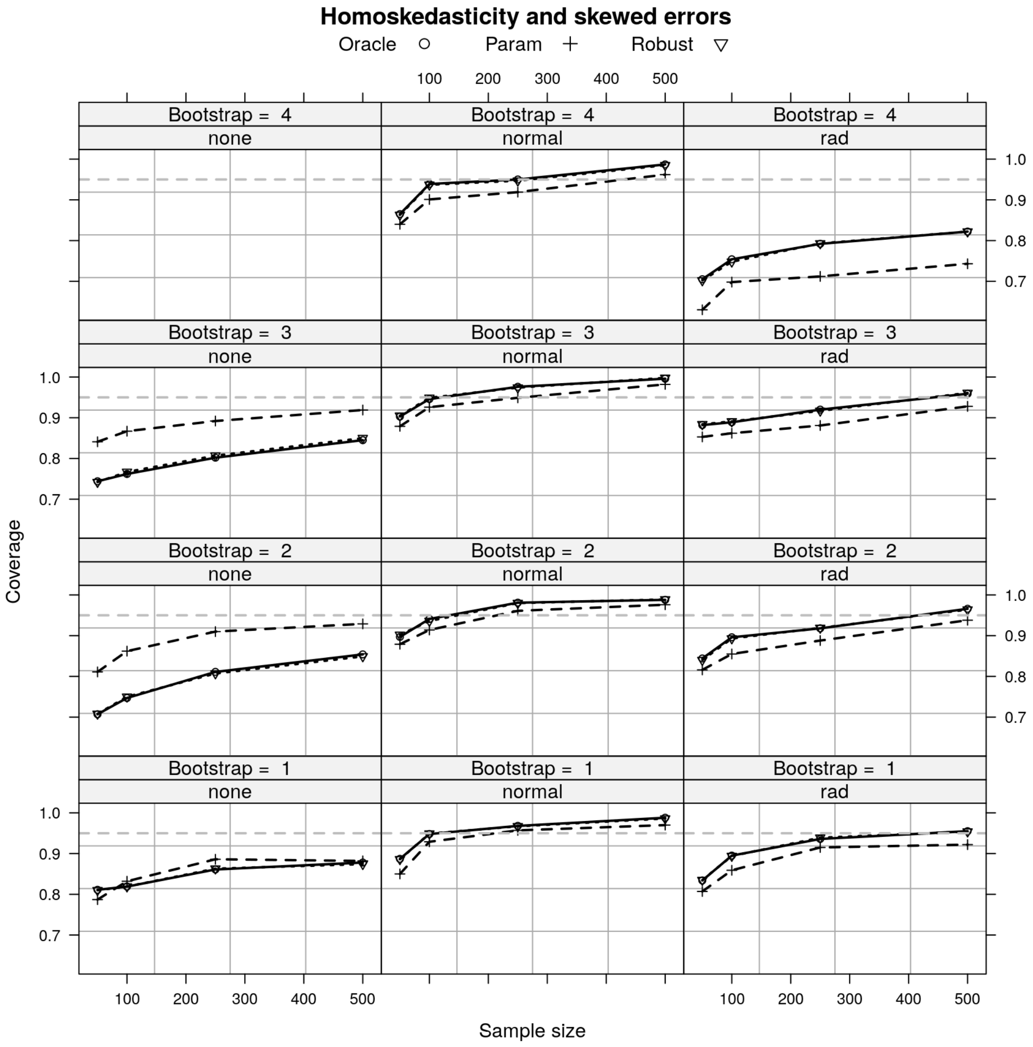

Homoskedasticity and skewed errors
Oracle
Param
Robust
Bootstrap = 4
none
normal
rad
Bootstrap = 3
Bootstrap = 2
Bootstrap = 1
Coverage
Sample size
100
200
300
400
500
0.7
0.8
0.9
1.0

$S = 0.66$

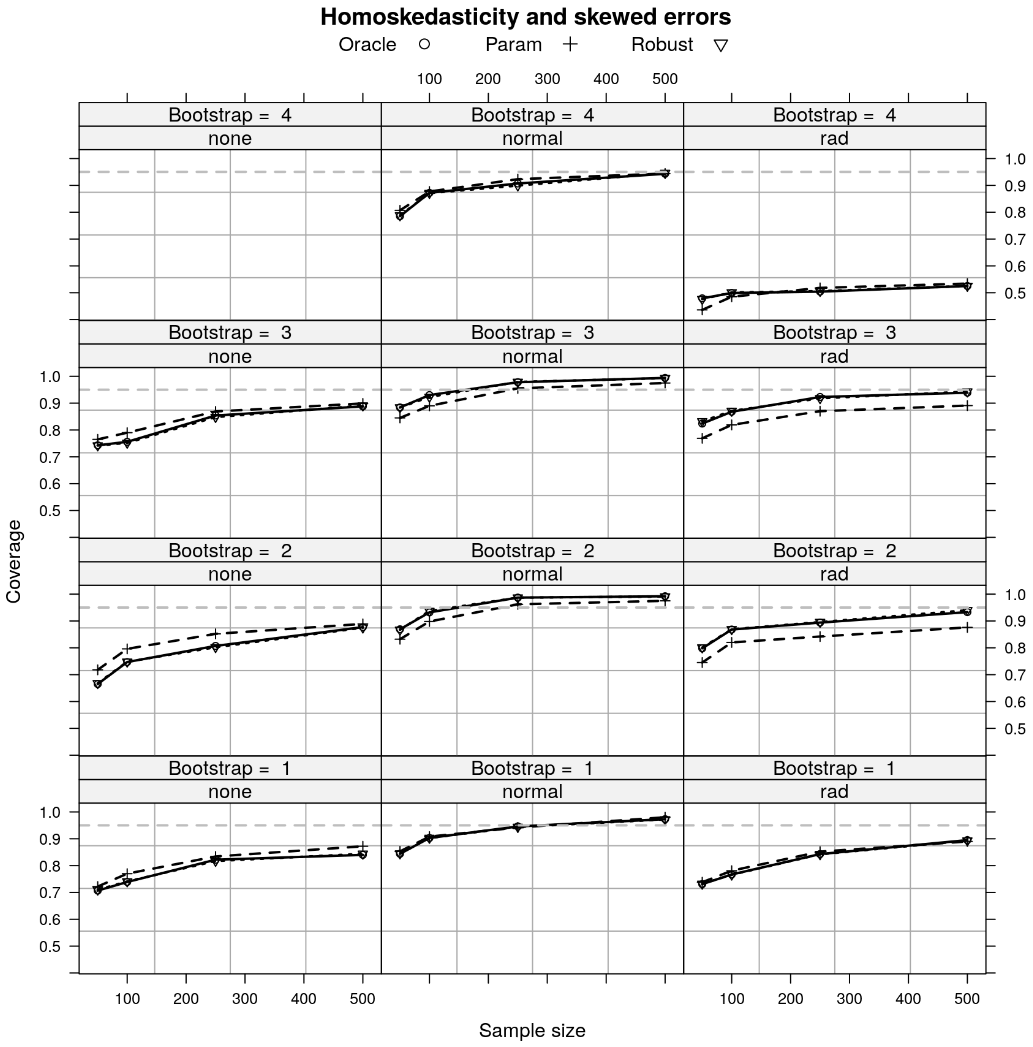
Homoskedasticity and skewed errors
Oracle
Param
Robust
Bootstrap = 4
Bootstrap = 3
Bootstrap = 2
Bootstrap = 1
none
normal
rad
Coverage
Sample size
100
200
300
400
500
0.5
0.6
0.7
0.8
0.9
1.0

$S = 1$

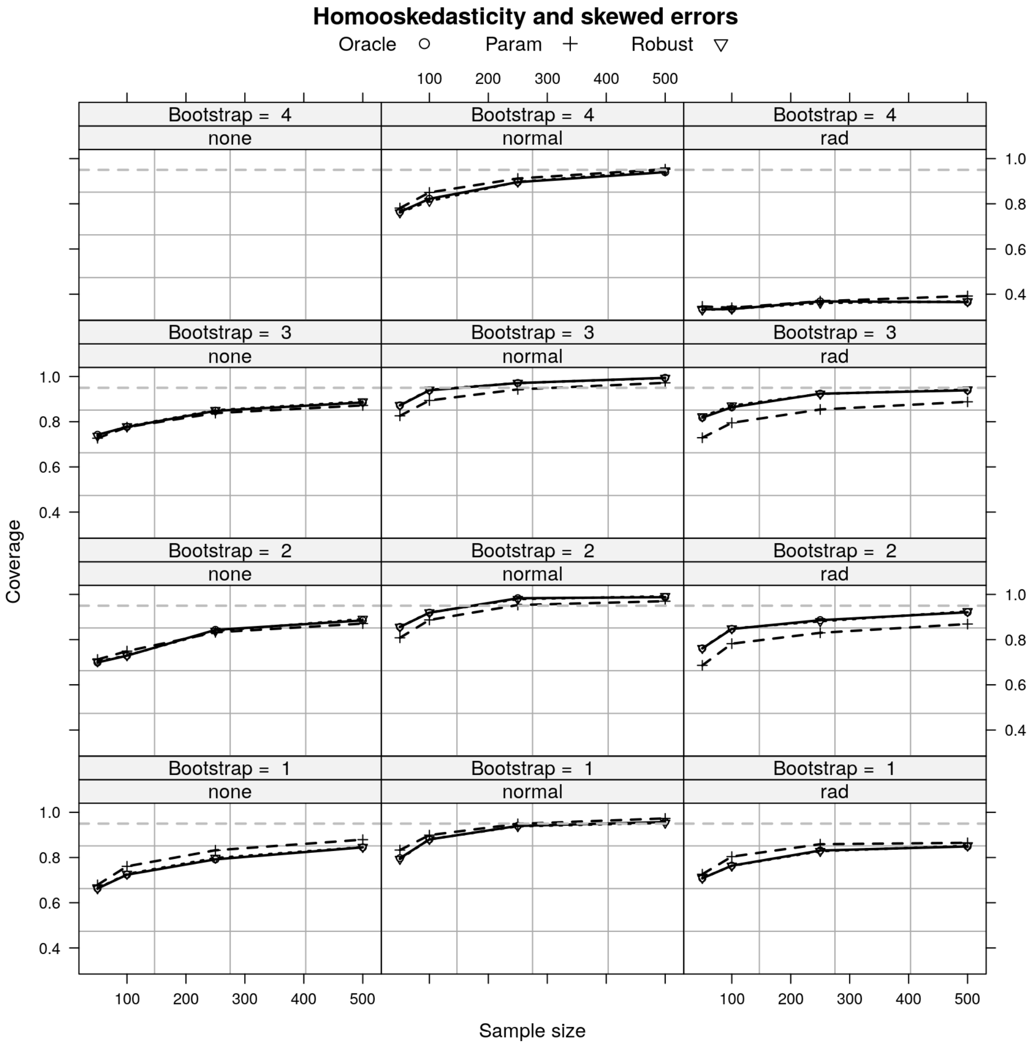

Homooskedasticity and skewed errors
Oracle Param Robust
Bootstrap = 4
none
normal
rad
Bootstrap = 3
Bootstrap = 2
Bootstrap = 1
Coverage
Sample size
100 200 300 400 500
0.4 0.6 0.8 1.0

Heteroskedasticity & Skewed Errors

$S = 0$

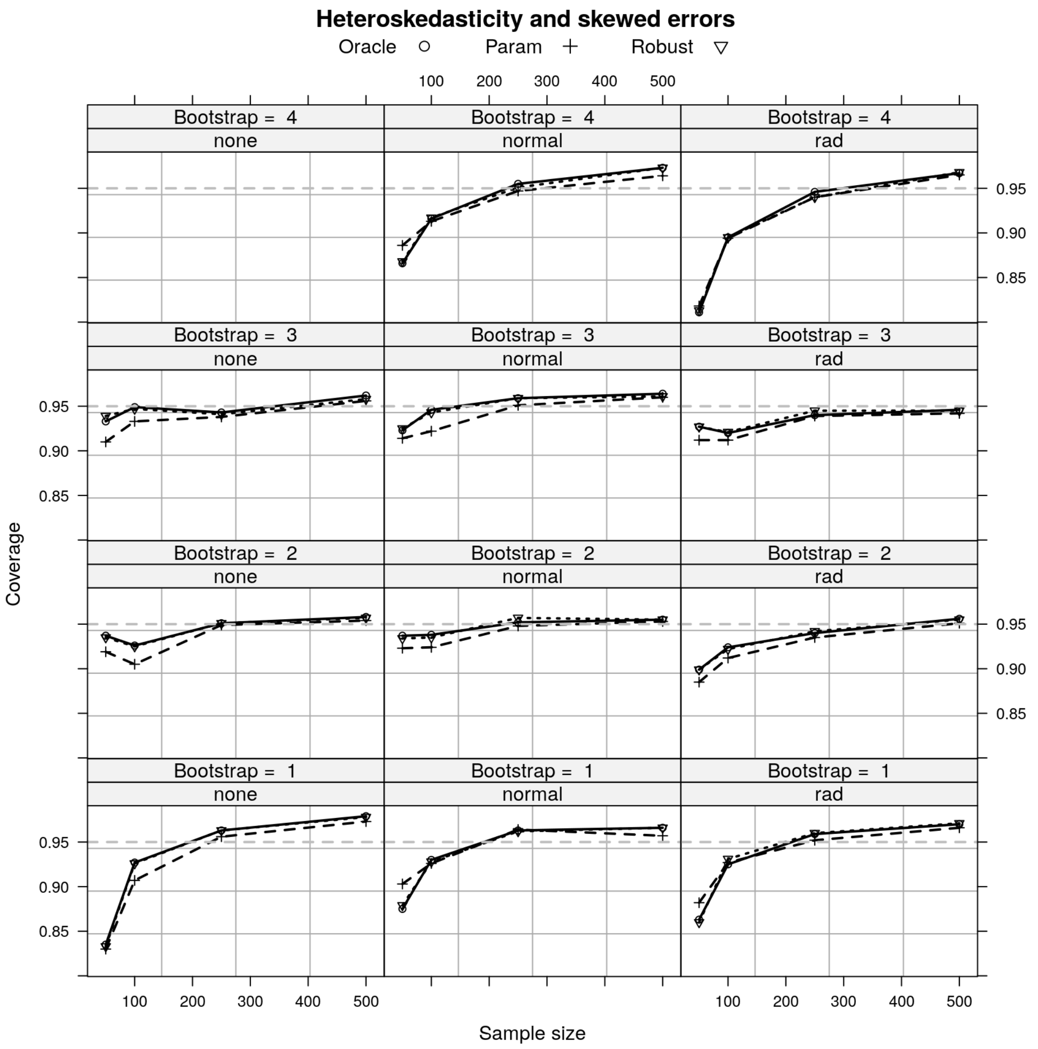

$S = 0.33$

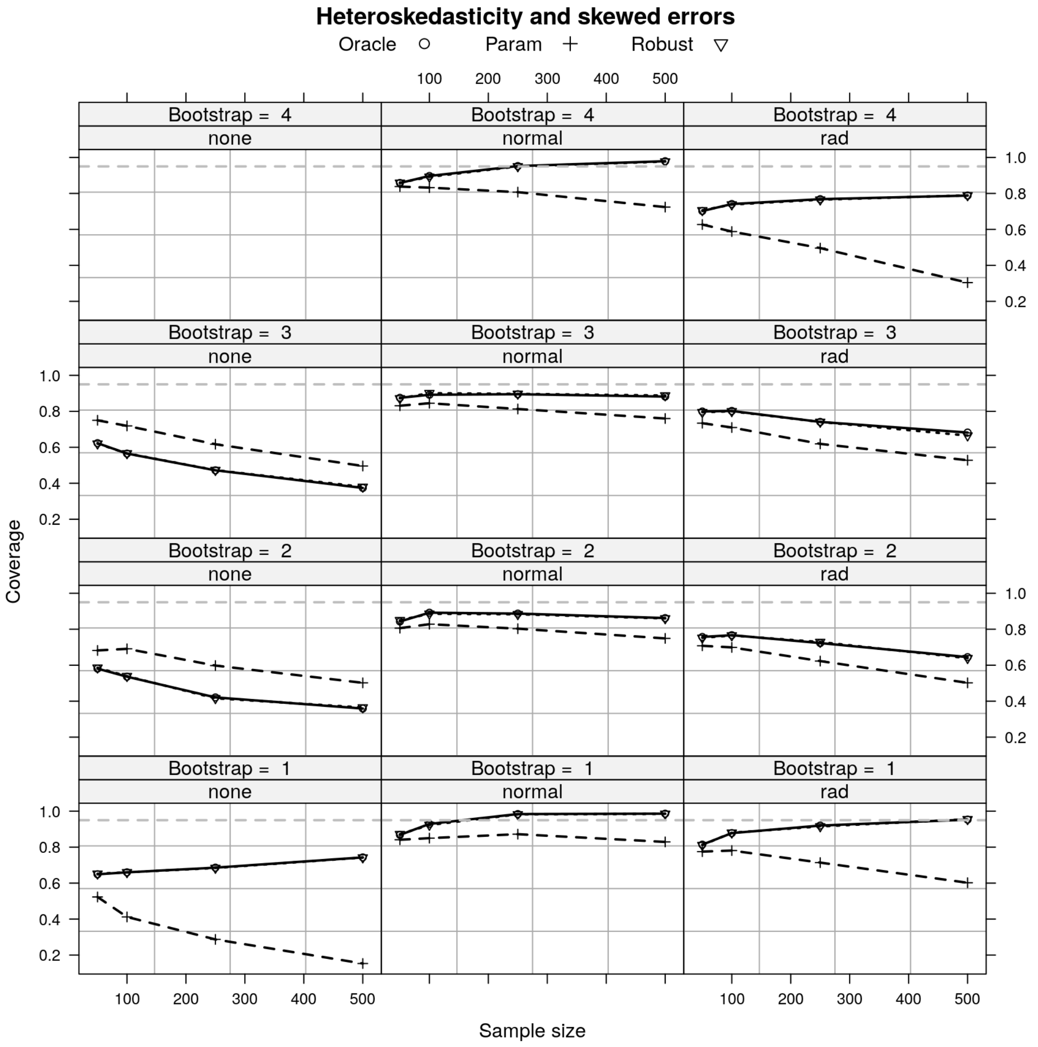

Heteroskedasticity and skewed errors
Oracle
Param
Robust
Bootstrap = 4
Bootstrap = 3
Bootstrap = 2
Bootstrap = 1
none
normal
rad
Coverage
Sample size
100
200
300
400
500
0.2
0.4
0.6
0.8
1.0

$S = 0.66$

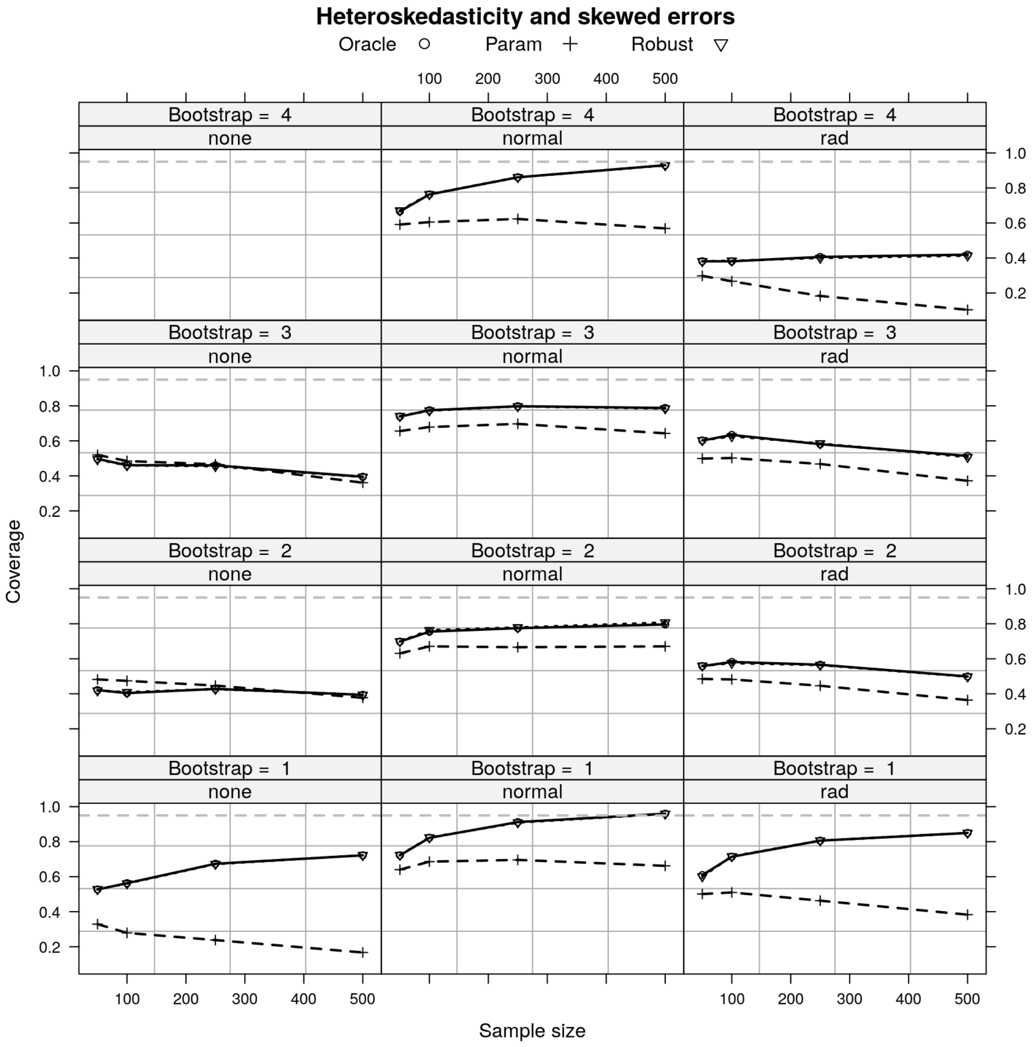

Heteroskedasticity and skewed errors
Oracle
Param
Robust
Bootstrap = 4
none
normal
rad
Bootstrap = 3
Bootstrap = 2
Bootstrap = 1
Coverage
Sample size
100
200
300
400
500
0.2
0.4
0.6
0.8
1.0

$S = 1$

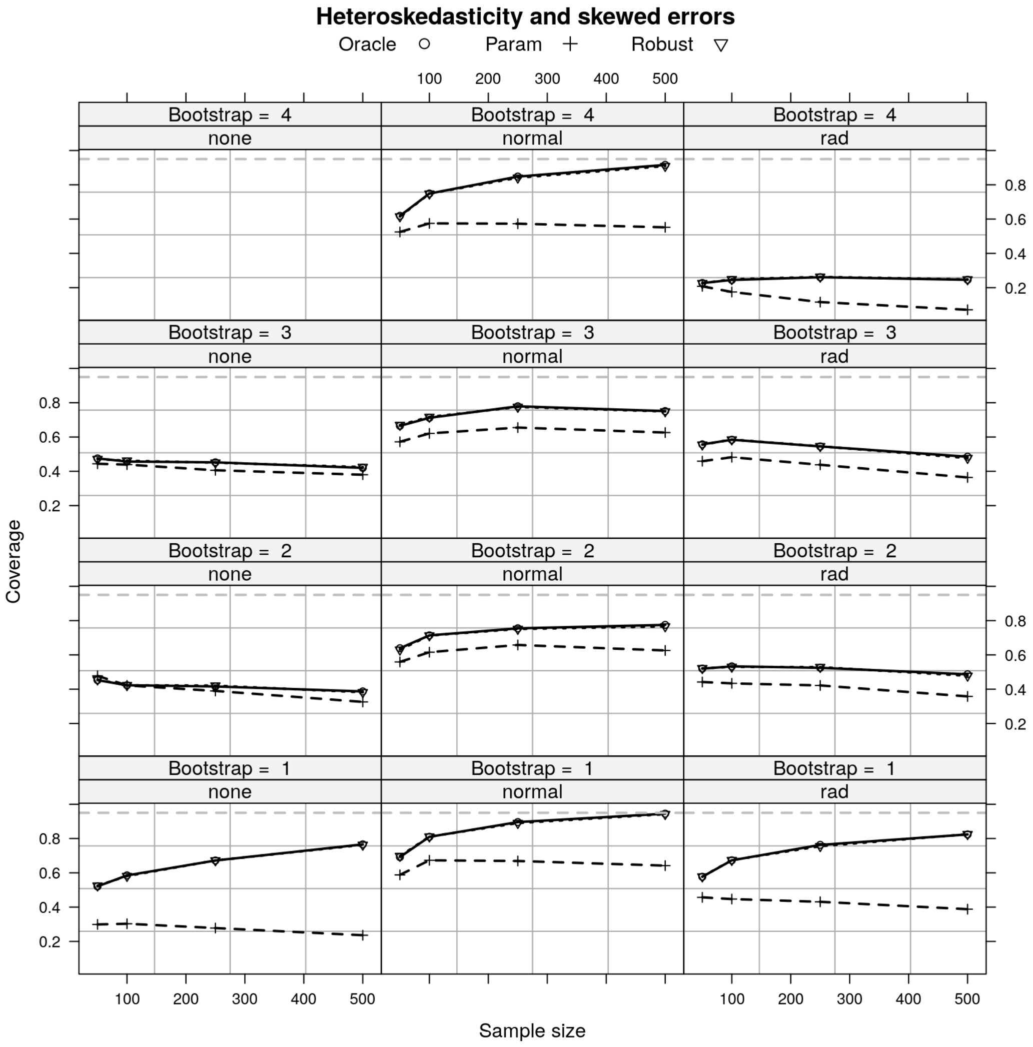

Heteroskedasticity and skewed errors
Oracle
Param
Robust
Bootstrap = 4
Bootstrap = 3
Bootstrap = 2
Bootstrap = 1
none
normal
rad
Coverage
Sample size
0.2
0.4
0.6
0.8
100
200
300
400
500

References

Wu, C. F. J. (1986). Jackknife, Bootstrap and Other Resampling Methods in Regression Analysis. *The Annals of Statistics*, 14(4):1261–1295. Publisher: Institute of Mathematical Statistics.
